# Automated Prediction of Glasgow Coma Scale Scores From Unstructured Electronic Health Records Using Natural Language Processing: Development and Validation Study

**DOI:** 10.2196/81245

**Published:** 2026-06-29

**Authors:** Marta Fernandes, Niels Turley, Haoqi Sun, Shibani S Mukerji, Lidia M V R Moura, M Brandon Westover, Sahar F Zafar

**Affiliations:** 1 Department of Neurology, Massachusetts General Hospital, 55 Fruit St, Boston, MA, 02114, United States, 1 8573319160; 2 Department of Neurology, Beth Israel Deaconess Medical Center, Boston, MA, United States

**Keywords:** Glasgow Coma Scale, electronic health records, phenotyping, natural language processing, machine learning

## Abstract

**Background:**

Multicenter electronic health records (EHRs) can support quality improvement and comparative effectiveness research in critical care. However, limitations of EHR-based research include challenges in abstracting key clinical variables, including a patient’s level of consciousness.

**Objective:**

This study aimed to develop a natural language processing model to predict Glasgow Coma Scale (GCS) scores from daily EHR notes.

**Methods:**

The study included adult patients (aged ≥18 years) admitted to Mass General Brigham (MGB) hospitals (2017‐2024) and patients from the Medical Information Mart for Intensive Care-III (MIMIC-III version 1.4; 2001‐2012) database. A dataset of all patients from both institutions was split into training (70%) or hold-out test (30%) sets. Variables consisted of daily notes, age, sex, and admission type. A pooled ordinal regression model (ordinalNet) with an elastic net penalty was trained to predict the lowest daily level of consciousness across 3 classes of impairment: severe (GCS score 3‐8), moderate (GCS score 9‐12), and mild (GCS score 13‐15), and a pooled linear model was trained to predict continuous GCS scores (3-15). Gold standard GCS was obtained from structured flowsheet data. External generalizability was assessed using a single-institution ordinal model trained on MGB and tested on MIMIC. Following post hoc calibration, the performance of the ordinal and linear models was evaluated on the hold-out test sets using the area under the receiver operating characteristic curve (AUROC) and area under the precision-recall curve (AUPRC) for the ordinal models and root mean square error and Pearson correlation coefficient for the linear models.

**Results:**

The modeling cohort included 145,897 patients (MGB: n=123,257, MIMIC: n=22,640), with 1,446,965 days of hospitalization between training and testing sets; the average age was 62 (SD 18) years, and the sex distribution was balanced. The pooled ordinalNet achieved an AUROC of 0.96 (95% CI 0.96‐0.96) and an AUPRC of 0.77 (95% CI 0.76‐0.77). The single-institution ordinal model achieved an AUROC of 0.90 (95% CI 0.89‐0.90) and an AUPRC of 0.80 (95% CI 0.79‐0.80). The pooled linear model achieved a root mean square error of 2.30 (95% CI 2.30‐2.30) and a correlation of 0.76 (95% CI 0.76‐0.76). Predictions for severe GCS were driven by terms indicating unresponsiveness and critical interventions, moderate GCS by intermediate alertness descriptors, and mild GCS by mentions of normal or awake behavior.

**Conclusions:**

Pooled ordinal and linear models can accurately predict GCS from unstructured data and can support large-scale phenotyping of neurological assessments for future critical care research.

## Introduction

The Glasgow Coma Scale (GCS) is used widely to characterize the level of consciousness in critically ill medical, surgical, neurological, and trauma patients [1]. It is a key component of most critical illness disease severity scores, including the Acute Physiology and Chronic Health Evaluation II score, the Sequential Organ Failure Assessment score, and the Simplified Acute Physiology score [2-4]. It is also used to define disease severity in patients with traumatic brain injuries [5,6]. GCS correlates strongly with morbidity and mortality in critically ill patients and in patients with traumatic brain injuries [7,8]. Therefore, GCS serves as a key variable in critical care research studies, quality improvement initiatives, epidemiological studies, and population health research [9,10]. However, GCS is often missing in electronic health records (EHRs), posing challenges in defining illness severity and calculating key illness severity scores [11]. GCS is also frequently missing in trauma studies [12]. Often, imputation methods are used to account for missing GCS data in calculating critical illness severity scores [13,14]. In addition, GCS frequently fluctuates, and documented scores may not capture the fluctuations [14]. Finally, while GCS is commonly documented in structured flowsheets, often greater details on component scores and fluctuations are provided in unstructured EHR data [15].

The goal of this study was to develop an automated natural language processing (NLP) model capable of estimating GCS scores from unstructured clinical text, allowing inference of neurological status in clinical notes where structured GCS documentation is incomplete or missing. This can support assessment of disease severity in EHR-based population health science research and quality improvement and comparative effectiveness studies, as well as cohort identification for clinical trials in patients with neurologic injury or disorders of consciousness.

## Methods

### Study Population

We included adult patients (aged ≥18 years) with an inpatient hospital admission. This study consists of retrospective data analysis and is reported in accordance with the Transparent Reporting of a Multivariable Prediction Model for Individual Prognosis or Diagnosis–Artificial Intelligence (TRIPOD-AI) statement [16].

### Ethical Considerations

The study was approved by the Mass General Brigham (MGB) Institutional Review Board (protocol 2013P001024). A waiver of informed consent was obtained for this observational study.

### Datasets

Our cohort was derived from two sources: (1) inpatient admissions to the MGB health care system between January 2017 and May 2024 and (2) admissions recorded in the publicly available, deidentified Medical Information Mart for Intensive Care-III (MIMIC-III) database, version 1.4 [17], which contains medical records for intensive care unit admissions at Beth Israel Deaconess Medical Center between 2001 and 2012. The cohort included adult patients with daily clinical notes and at least 1 daily assessment of the total GCS score.

### Clinical Variables

Structured variables included age, sex, and admission types (emergency, urgent, and elective). Text-based variables were extracted from preprocessed daily clinical notes and binarized (Multimedia Appendix 1). MGB notes included procedures, nursing, case management, consults, assessment and plan, history and physical, progress, physical therapy, occupational therapy, and discharge summaries. MIMIC notes included electrocardiogram, echocardiogram, radiology, respiratory, nutrition, general, physician, nursing, rehab services, case management, consult, and discharge summaries. For both institutions, inpatient days with only the following note types, either alone or combined, were removed: procedures, radiology, electrocardiogram, echocardiogram, respiratory, and nutrition. These note types were considered to contain minimal information regarding the patient’s neurological state.

To avoid conflating pathologic coma with iatrogenic sedation and enhance clinical validity, text features containing sedative and anesthetic medications (eg, propofol or other sedatives or anesthetics) were removed, ensuring the model focused on physiological descriptions of consciousness.

### Outcomes and Gold Standard

Our outcome was the lowest daily GCS score for each day of hospital admission. For analysis, the lowest daily GCS score was categorized as severe (GCS score 3‐8), moderate (GCS score 9‐12), and mild (GCS score 13‐15). For both MGB and MIMIC cohorts, the gold standard scores were obtained from structured information tables.

### Statistical Analysis

Data from each institution was randomly split by patient into training (70%) and hold-out test (30%) sets. The training sets from both MGB and MIMIC, as well as their respective test sets, were combined to create pooled training and testing sets for a single, multi-institution model. A combination of undersampling and oversampling strategies was applied within the training set to prevent 1 institution from dominating the pooled model and to address class imbalance and mitigate potential bias toward the majority class (Multimedia Appendix 1).

### Model Design

We developed an ordinal regression model with elastic net penalty [18] (ordinalNet) within the training data to predict the 3 classes of GCS scores: mild (13-15), moderate (9-12), and severe (3-8). We also developed a linear regression model to predict the full range of GCS scores: 3‐15. Data were split into train and test and resampled similar to the ordinal regression (Multimedia Appendix 1).

We additionally evaluated cross-institution generalizability by training a single-institution model using data from MGB and testing it on MIMIC data (Multimedia Appendix 1). This approach allowed us to assess the generalizability of models trained on data from a single center compared with models trained on multicenter data.

### Model Evaluation

For model evaluation, we used the area under the receiver operating characteristic curve (AUROC), area under the precision-recall curve (AUPRC), sensitivity (or recall), specificity, positive predictive value, negative predictive value, and *F*_1_-score. We present the macroaverage performance, which calculates each metric independently for each class and then takes the average.

Calibration was performed post hoc using multinomial logistic regression fitted on the model-predicted GCS class probabilities. The calibration model was fit on the training data only and evaluated on the hold-out test set. Calibration was evaluated using reliability diagrams comparing predicted and observed class probabilities. Model calibration was assessed using both the multiclass Brier score, which treats each class as nominal, and the ordinal Brier score, which accounts for the ordering of the classes. A lower Brier score indicates better calibrated predictions.

A total of 1000 bootstrap iterations with random sampling and replacement were performed to calculate 95% CIs for performance metrics. We report overall results for the pooled institution model on the combined hold-out test set and separately by institution. We additionally report results for the single-institution model.

## Results

### Patients’ Characteristics

**Figure 1.**
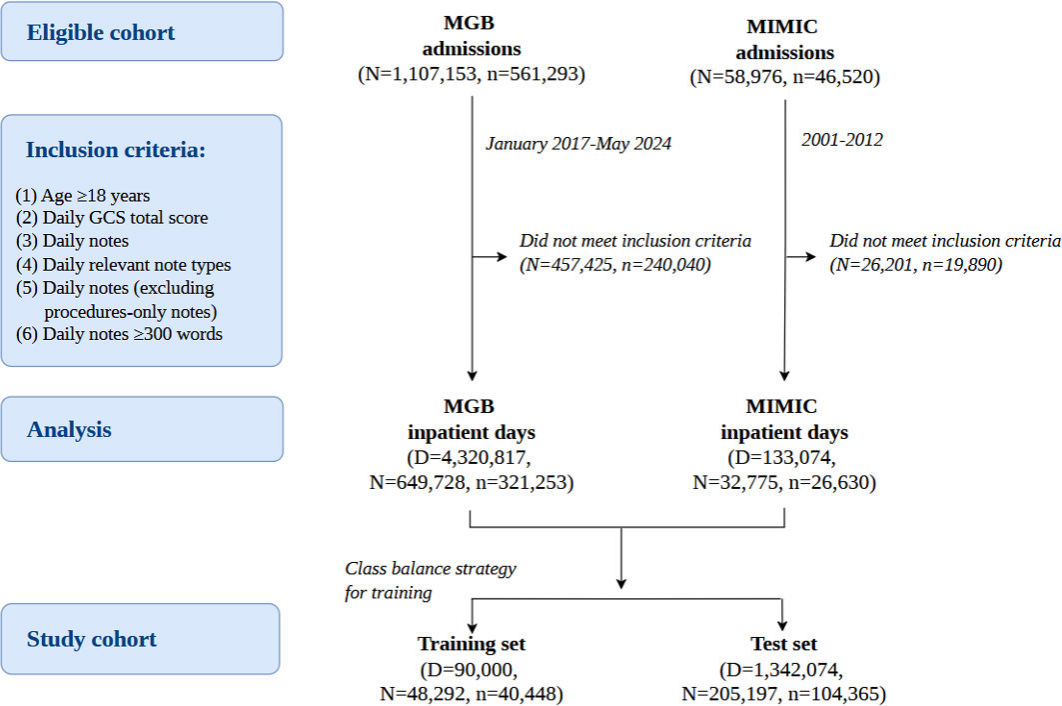
CONSORT-AI (Consolidated Standards of Reporting Trials–Artificial Intelligence) flow diagram. The number of patients is represented by ‘“n,” the number of hospital admissions by “N,” and the number of daily inpatient stays by “D.” GCS: Glasgow Coma Scale; MGB: Mass General Brigham; MIMIC: Medical Information Mart for Intensive Care.

Our study cohort included 145,897 patients (MGB=123,257, MIMIC=22,640), with a total of 1,446,965 days of inpatient admissions. The average age of patients in our study cohort was 62 (SD 18) years, and the cohort had a balanced sex distribution (n=75,866, 52%). The patients' race and ethnicity distribution is shown in Table 1. These variables were collected for descriptive purposes and not used for analysis. Overall, the multicenter train and hold-out test sets were balanced at the patient level, with a majority of emergency admissions in both sets. After applying inclusion and exclusion criteria (Figure 1), we observed a higher proportion of exclusion in the MGB data for patients who did not present all 3 GCS components—visual, verbal, and motor—thus not a total GCS score. In the MIMIC data, a higher proportion of exclusion was for inpatient days including only procedure notes. To improve readability, the flow diagram reports total numbers after exclusion criteria, while detailed exclusion counts at patient and admission levels by criterion are provided in Table S2 in Multimedia Appendix 1. We performed a sensitivity analysis for notes length threshold and selected a minimum of 300 words per day for model training (Tables S3 and S4 in Multimedia Appendix 1).

We observed that MGB test data are heavily skewed toward mild cases (n=116,028, 89%), with very few severe or moderate cases. MIMIC test data are more balanced, with substantially higher proportions of moderate and severe cases than the MGB test data (Table 1).

**Table 1.** Characteristics of the study population.

Characteristic	Training set			Testing set		
	MGB^a^ (n=26,718)	MIMIC^b^ (n=14,022)	Total (n=40,740)	MGB (n=96,539)	MIMIC (n=8618)	Total (n=105,157)
Age (years), mean (SD)	64 (17)	64 (17)	64 (17)	62 (18)	63 (18)	62 (18)
Sex (male), n (**%**)	14,598 (55)	7954 (57)	22,552 (55)	48,784 (51)	4827 (56)	53,611 (51)
Race^c^, n (%)						
Asian	931 (3)	315 (2)	1246 (3)	3063 (3)	186 (2)	3249 (3)
Black or African American	2354 (9)	1043 (7)	3397 (8)	6914 (7)	642 (7)	7556 (7)
Hispanic or Latino	2127 (8)	423 (3)	2550 (6)	7876 (8)	271 (3)	8147 (8)
White	20,567 (77)	10,175 (73)	30,742 (76)	77,286 (80)	6238 (72)	83,524 (80)
Other^d^	2866 (11)	2489 (18)	5355 (13)	9276 (10)	1552 (18)	10,828 (10)
Hospital daily stays^e^, n	45,000	45,000	90,000	1,308,700	48,265	1,356,965
Same day death^e^^f^, n (%)	764 (2)	917 (2)	1681 (2)	4265 (0.3)	1048 (2)	5313 (0.4)
Admission type^e^, n (%)						
Emergency	28,933 (64)	38,344 (85)	67,277 (75)	865,201 (66)	40,931 (85)	906,132 (67)
Urgent	9048 (20)	1693 (4)	10,741 (12)	202,175 (15)	5478 (4)	207,653 (15)
Elective	8093 (18)	4963 (11)	13,056 (15)	255,523 (20)	4029 (11)	259,552 (19)
GCS^e^^g^ classes, n (%)						
Severe (3-8)	15,000 (33)	15,000 (33)	30,000 (33)	64,891 (5)	15,407 (32)	80,298 (6)
Moderate (9-12)	15,000 (33)	15,000 (33)	30,000 (33)	83,526 (6)	13,036 (27)	96,562 (7)
Mild (13-15)	15,000 (33)	15,000 (33)	30,000 (33)	1,160,283 (89)	19,822 (41)	1,180,105 (87)

a MGB: Mass General Brigham.

b MIMIC: Medical Information Mart for Intensive Care.

c Race, ethnicity, and same day death are presented for descriptive purposes to characterize the cohort; these variables were not used as predictors in the modeling analyses.

d Includes American Indian or Alaska Native, multirace, and unknown race.

e Number of daily inpatient stays.

f Death on the same day of inpatient day.

g GCS: Glasgow Coma Scale.

### Pooled Ordinal Model Performance

**Figure 2.**
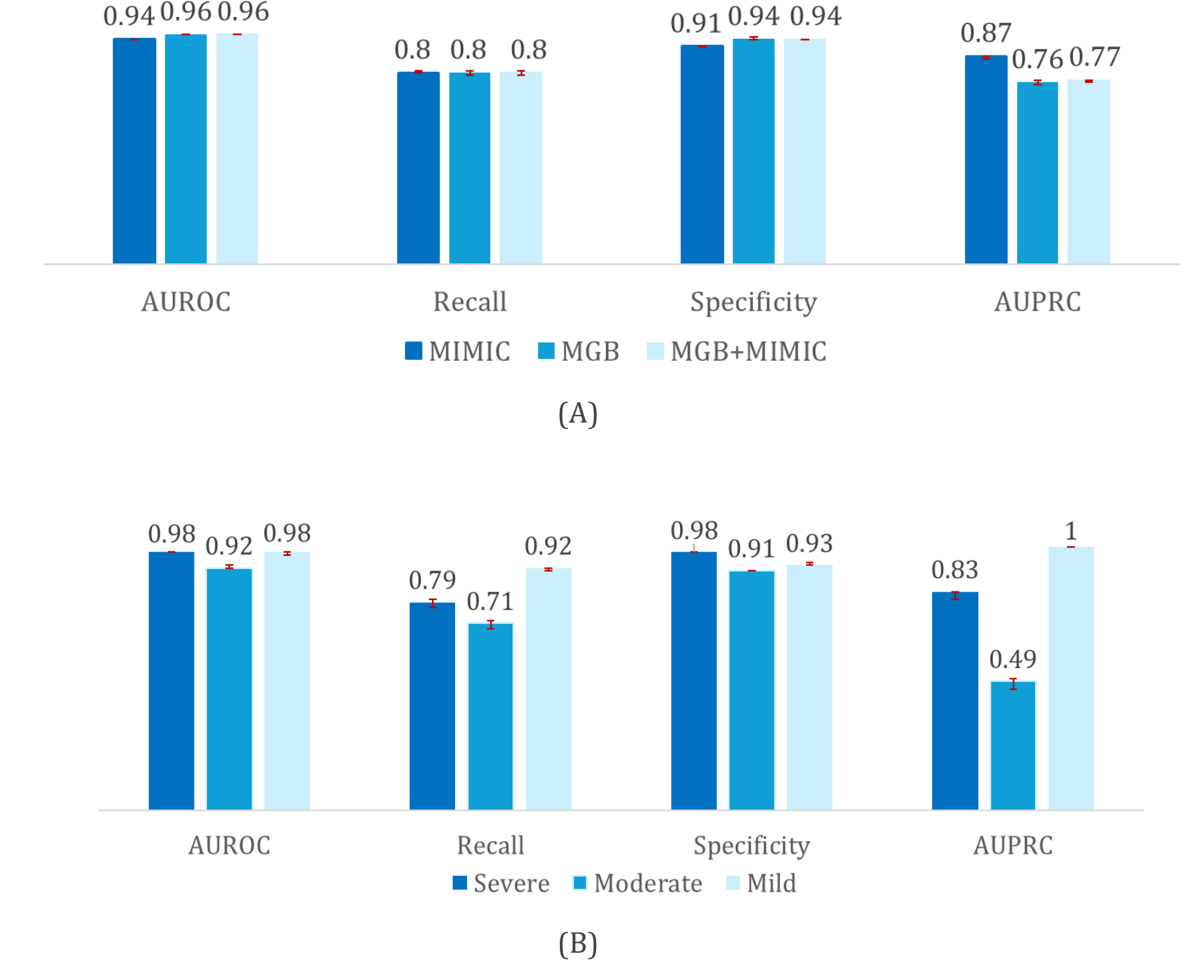
Macroaverage performance of the pooled model on the hold-out test set for the Glasgow Coma Scale prediction: (A) across combined ordinal classes and (B) stratified by individual ordinal classes AUROC: area under the receiver operating characteristic; AUPRC: area under the precision-recall curve; MGB: Mass General Brigham; MIMIC: Medical Information Mart for Intensive Care.

**Figure 3.**
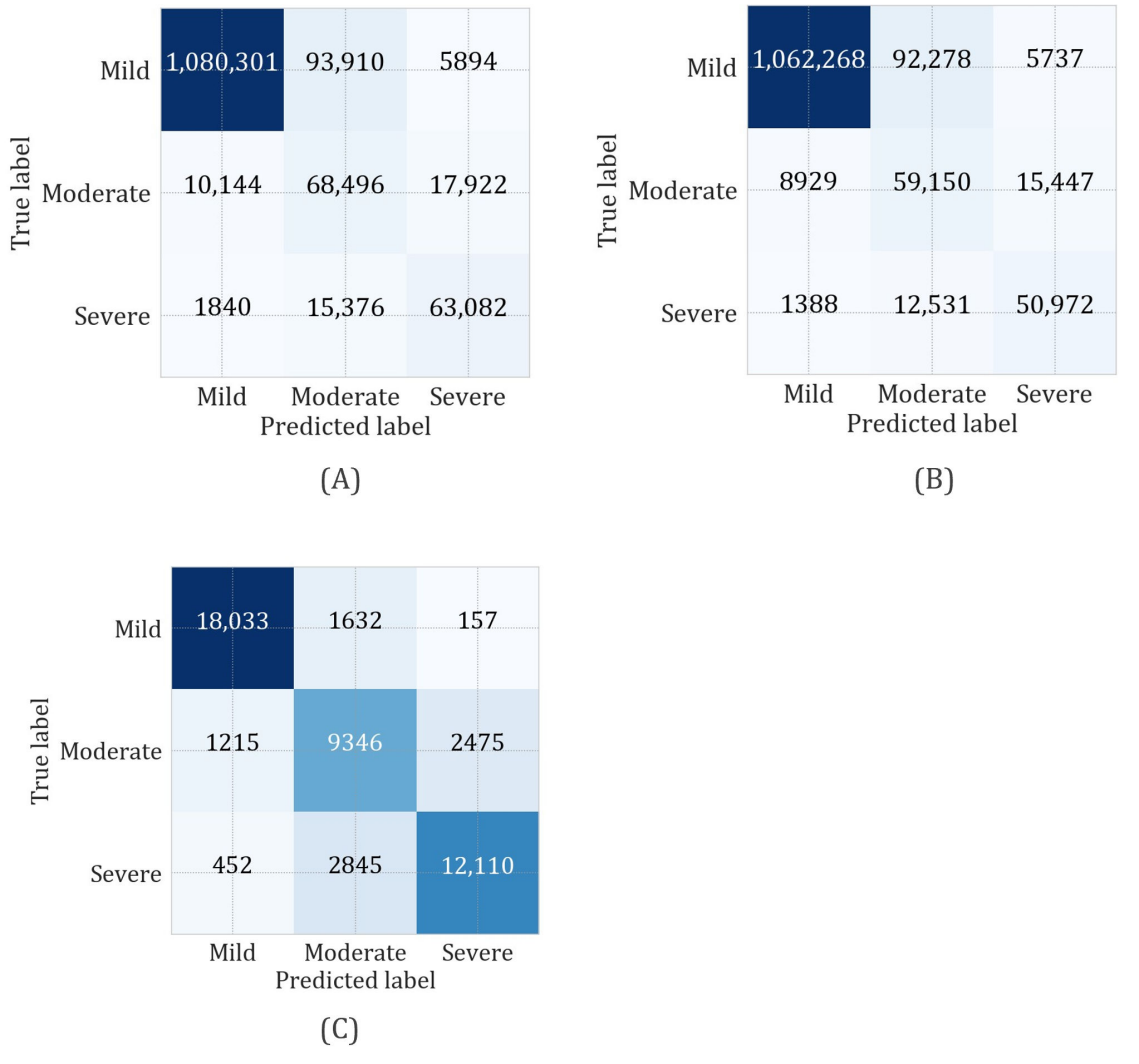
Confusion matrices of the pooled model on the hold-out test set: (A) full test set, (B) Mass General Brigham, and (C) Medical Information Mart for Intensive Care.

Performance of the pooled model was evaluated separately for overall multicenter MGB+MIMIC data and separately for each institution using postcalibration predictions. The overall pooled ordinal model achieved an AUROC of 0.96 (95% CI 0.96‐0.96) and an AUPRC of 0.77 (95% CI 0.76‐0.77) after calibration (Figure 2). When evaluated on each institution test set, the model achieved a combined recall of 0.80 (95% CI 0.79‐0.81; for MGB+MIMC), while MIMIC achieved an AUPRC of 0.87 (95% CI 0.86‐0.87), 10% higher than MGB (0.76, 95% CI 0.75‐0.77). Confusion matrices revealed that most misclassification occurred in the moderate class (Figure 3). MGB was heavily skewed toward mild cases (n=116,028, 89% of inpatient days), as seen in Table 1, resulting in the majority of predictions being correctly classified as mild, with relatively few moderate and severe cases. MIMIC had a more balanced distribution across mild, moderate, and severe classes, which led to a higher absolute number of misclassifications between moderate and severe, despite comparable proportional performance. These differences in the confusion matrices are therefore largely explained by the underlying class distributions in the test sets rather than differences in model performance.

The pooled ordinal model’s AUROC and AUPRC plots are presented in Figures S1 to S3 in Multimedia Appendix 1, macroaveraged performance across all metrics before and after calibration are reported in Tables S6 and S7 in Multimedia Appendix 1, and the modeling parameters are provided in Table S8 in Multimedia Appendix 1. All performance metrics reported here (area under the curve, recall, specificity, AUPRC, and confusion matrices) are based on postcalibration predictions.

### Ordinal Model Calibration

**Figure 4.**
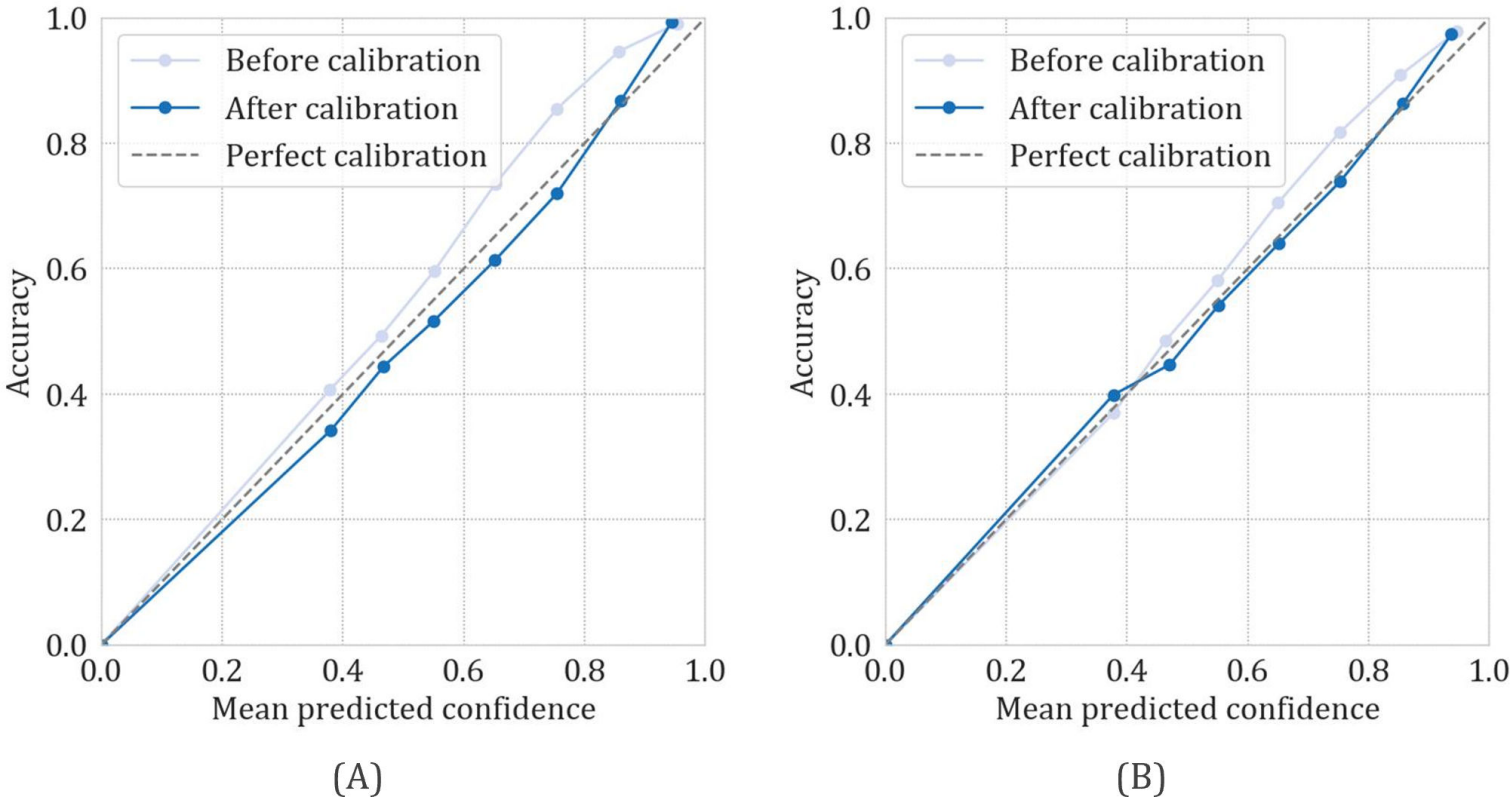
Calibration of ordinal class probabilities on the hold-out test set: institution-specific reliability diagrams for (A) Mass General Brigham and (B) Medical Information Mart for Intensive Care.

We assessed the reliability of calibration per institution, as shown in Figure 4. Prior to calibration, the model is systematically overconfident at higher predicted probabilities. After calibration, predicted probabilities are better aligned with observed accuracies, indicating improved probabilistic calibration. For MIMIC, calibration curves align more closely with the ideal diagonal across the full probability range. Calibration produced minimal changes in class-specific performance metrics (Tables S6 and S7 in Multimedia Appendix 1), suggesting that the original ordinalNet probabilities were already well calibrated.

We also evaluated Brier scores before and after calibration and observed that the ordinal Brier score for the pooled ordinal model remained unchanged, with a value of 0.10 (95% CI 0.09‐0.10). Institution-specific Brier scores are reported in Table S9 in Multimedia Appendix 1.

### Single-Institution Ordinal Model Performance

The single-institution model was trained on MGB train set data (number of daily inpatient stays=45,000, n=26,718; Table 1) and externally evaluated on the MIMIC full test set (number of daily inpatient stay=160,375, n=28,725). The model achieved an AUROC of 0.90 (95% CI 0.89‐0.90), a recall of 0.72 (95% CI 0.71‐0.72), a specificity of 0.87 (95% CI 0.86‐0.87), and an AUPRC of 0.80 (95% CI 0.79‐0.80). Performance results for each ordinal label are also presented in Tables S6 and S7 in Multimedia Appendix 1. AUROC and AUPRC plots, as well as the confusion matrix, are shown in Figure S4 in Multimedia Appendix 1.

Post hoc multinomial calibration slightly reduced discrimination (recall 0.73 vs 0.72; Tables S6 and S7 in Multimedia Appendix 1) and slightly increased Brier error (0.23 vs 0.24; Table S8 in Multimedia Appendix 1), suggesting that the original ordinalNet probabilities were already well calibrated within the institutional distribution.

### Linear Model Performance

We evaluated the performance of the linear model on the same hold-out test set as the ordinal model. Details of cohort derivation are presented in the CONSORT-AI (Consolidated Standards of Reporting Trials–Artificial Intelligence) modeling charts in Figure S5 in Multimedia Appendix 1. The pooled linear model achieved a Pearson correlation of 0.76 (root mean square error [RMSE] 2.40, 95% CI 2.40-2.41) on the hold-out test set (Table S5 in Multimedia Appendix 1). When evaluated separately, Pearson correlation was 0.74 for MGB (RMSE 2.40, 95% CI 2.40-2.41) and 0.82 for MIMIC (RMSE 2.47, 95% CI 2.45-2.48). Post hoc calibration improved absolute prediction accuracy (RMSE 2.30, 95% CI 2.30-2.30), while the Pearson correlation coefficient remained unchanged, consistent with the invariance of correlation to linear transformations.

Pooled linear model calibrated predictions vs target GCS scores are presented in Figure S6 in Multimedia Appendix 1, and the corresponding calibration curve is shown in Figure S7 in Multimedia Appendix 1.

### Error Analysis

We conducted a detailed analysis of misclassifications to identify potential limitations and areas for improvement in our model.

One source of model error was occasional inconsistency between the time of gold standard GCS measurements and the actual worst neurological condition of patients. In some cases, patients who deteriorated rapidly and died on the same day had only a single GCS score of 15 recorded early in the day, creating a mismatch between the documented score and the patient’s final clinical state. We retained these cases in our analysis to maintain a clinically representative dataset, acknowledging that excluding such edge cases would introduce selection bias.

Variability in measurement frequency posed another challenge. Some inpatient days had only a single daily GCS measurement, while others had multiple assessments. We chose not to restrict our analysis based on the number of measurements to avoid introducing sampling bias, as patients with more frequent monitoring might represent a clinically distinct population.

For patients with fluctuating consciousness levels throughout the day, the GCS documented in clinical notes often did not correspond to the lowest recorded gold standard GCS score.

These findings highlight both the challenges in developing accurate GCS prediction models and the potential advantages of NLP-based approaches in capturing the nuanced clinical picture that may not be fully represented in structured data alone.

### Feature Importance

**Figure 5.**
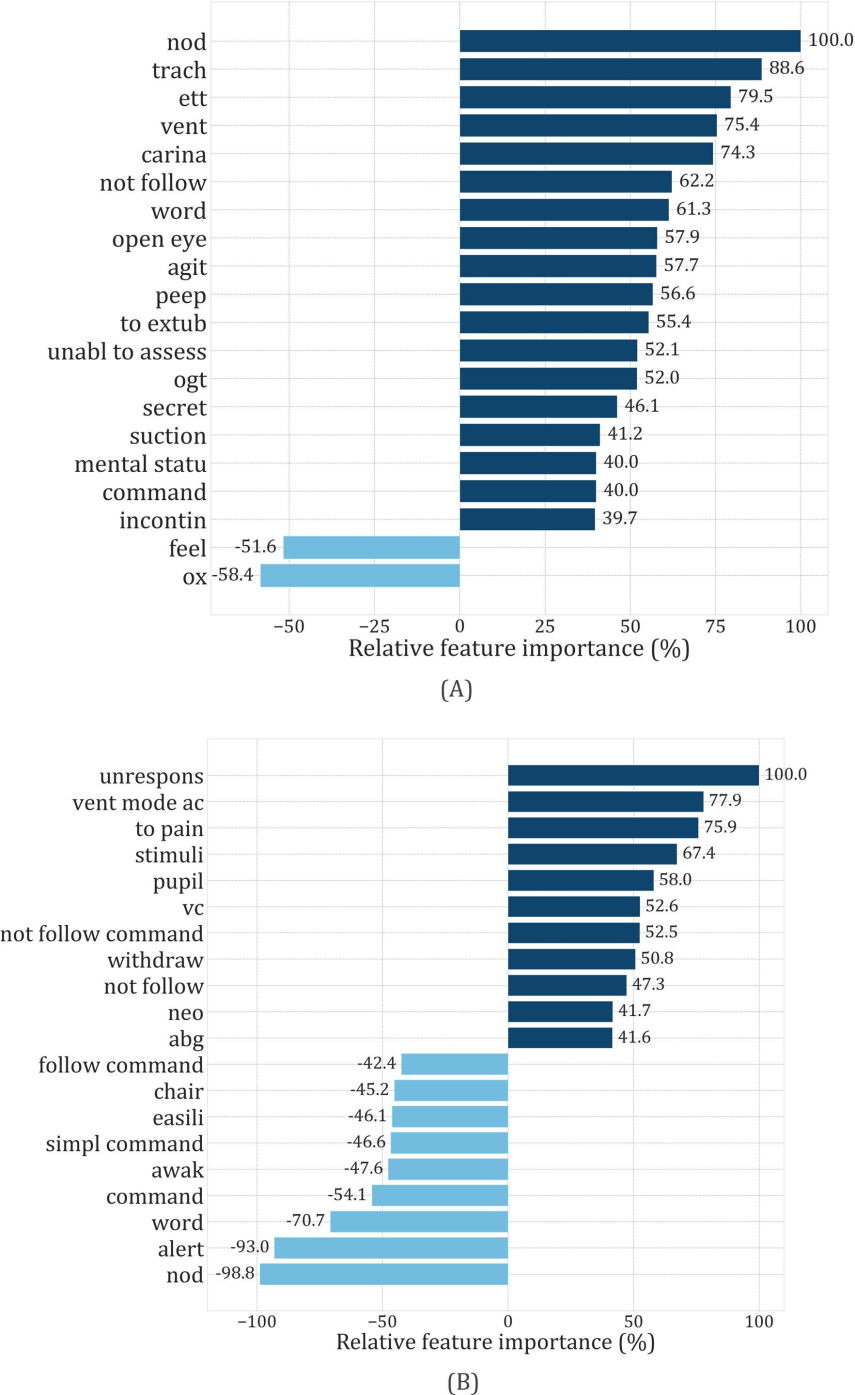
Feature importance from the pooled ordinal model for the top 20 features. (A) Threshold 1: mild vs moderate+severe and (B) threshold 2: mild+moderate vs severe. Positive coefficients indicate a higher likelihood of being above the threshold (more severe), while negative coefficients indicate a higher likelihood of being below the threshold (less severe). Stemmed features derived from clinical documentation include airway and respiratory terms (ett: endotracheal tube; trach: tracheostomy; vent: mechanical ventilation; peep: positive end-expiratory pressure; to extub: to extubate; ogt: orogastric tube; suction; secret, secretions; carina; vc: vital capacity; abg: arterial blood gas), neurologic examination terms (unrespons: unresponsive; stimuli; incontin: incontinence; withdraw: withdrawal; to pain: no response to pain; pupil; not follow: not follow command; follow command: follows commands; simpl command or command: obeys simple commands; word: verbal response; feel: sensory response; nod; open eye; mental statu: mental status; agit: agitation; awak: awake; alert; ox: derived from ”A&Ox” [alert and oriented x]), and documentation and context terms (unabl to assess: unable to assess; neo: phenylephrine or vasopressor; chair: mobilized to chair).

After vectorization of the preprocessed notes and removal of highly correlated features, as well as any features referring to sedative or opioid medications (eg, propofol, fentanyl, morphine, hydromorphone, oxycodone, and lorazepam [Ativan]), sedation, or anesthesia, the final feature set comprised 2705 text-derived features. Inclusion of age, sex, and admission type resulted in a total of 2708 features. The pooled ordinal model trained with the elastic penalty selected 541 features as relevant (with nonnull coefficients). The top 20 most relevant features for each model threshold are shown in Figure 5. The pooled linear model exhibited a similar pattern of feature importance (Figure S8 in Multimedia Appendix 1).

Feature importance patterns across ordinal thresholds revealed a clinically coherent gradient of severity (Figure 5). Mild cases were characterized by preserved alertness, command-following ability, verbal output, and spontaneous oxygenation. Moderate cases were distinguished primarily by airway instrumentation and mechanical ventilatory support, along with reduced but present responsiveness (patient nodding). In contrast, severe cases were dominated by features reflecting neurologic unresponsiveness, including lack of response to stimuli, pupillary abnormalities, and inability to follow commands. These findings suggest that respiratory support differentiates mild from higher severity states, whereas profound neurologic dysfunction defines the transition to severe illness.

## Discussion

### Principal Findings

We developed an NLP-based algorithm to classify GCS scores from daily inpatient notes of an adult population. The algorithm was developed using data from 2 health care institutions: one with inpatient admissions to any hospital inpatient setting (MGB) and the other with inpatient admissions to critical care units (MIMIC). The pooled ordinal model achieved high discrimination for predicting GCS across all classes (AUROC 0.96, 95% CI 0.96‐0.96 and AUPRC 0.77, 95% CI 0.76‐0.77). The model reliably provides predictions even when GCS scores are not directly documented, enabling automated ordinal scoring in real-world EHRs with incomplete documentation.

To ensure fair comparison of predicted probabilities, all models were evaluated after identical post hoc calibration. Calibration had minimal impact on both linear and ordinal predictions, indicating that the models’ outputs were inherently well calibrated. The pooled linear model achieved an overall good performance (RMSE 2.30; Pearson correlation 0.76). The higher Pearson correlation observed when testing the pooled linear model on MIMIC should be interpreted in the context of its substantially smaller test set. Correlation-based metrics are sensitive to sample size and outcome variability, and smaller, less heterogeneous cohorts may yield higher correlation estimates. In contrast, the larger and more diverse test set from MGB provides a more conservative estimate of performance. Importantly, these differences do not indicate inferior model performance, as pooled results remained stable across institutions.

The model trained on a single institution and tested on an external institution showed overall lower performance, highlighting limited generalizability when models are developed using data from only 1 site. Thus, single-institution models may not generalize well to external data, underscoring the benefit of pooling data across multiple institutions.

### Comparison With Prior Work and Potential Applications of Our Model

To our knowledge, this is the first work using an NLP-based algorithm to predict total GCS scores from unstructured clinical notes. Previous approaches have largely relied on structured data elements or manual chart review, which limits scalability and introduces potential selection bias. Our approach offers several advantages, including the ability to process large volumes of clinical documentation efficiently and extract neurological status information that may not be captured in structured fields.

Research in critical care, neurocritical care, and acute and nonacute neurology heavily relies on the GCS score as a key variable to measure illness severity, frequently used as an important confounder for adjustment, and also to determine eligibility for inclusion into epidemiology research and comparative effectiveness research, as well as for recruitment into clinical trials [19-27]. Missing GCS data are frequently a barrier in research that relies on EHR data [28,29]. Our algorithm addresses a major gap by developing a robust and efficient method to measure GCS when it is missing to facilitate EHR-based research. Another potential use of the algorithm is in critical care and trauma outcome studies, as well as in intensive care units survivorship studies, where data on baseline GCS scores are critical to understand relation to outcomes and survivorship [28]. Finally, missing data are a common barrier to EHR-based quality improvement efforts, and our model has potential applications in quality improvement interventions that rely on documentation of GCS or level of consciousness [30-32].

While our model showed excellent performance in identifying cases of severe consciousness impairment, the discrimination between moderate (GCS: 9‐12) and mild (GCS: 13‐15) categories was less robust. This likely reflects the clinical reality that the distinction between these categories can be subtle, with significant overlap in the language used to describe patients at these levels of consciousness. The threshold-specific feature importance patterns from the ordinal model suggest that this reflects the graded clinical structure of neurologic decline. Features distinguishing mild from higher severity states were largely related to airway instrumentation and ventilatory support (eg, endotracheal tube, tracheostomy, mechanical ventilation, and positive end‐expiratory pressure), along with reduced responsiveness (eg, not following commands). In contrast, the transition from moderate to severe was driven predominantly by markers of profound neurologic dysfunction, including unresponsiveness, abnormal responses to stimuli, pupillary findings, and inability to follow commands. Conversely, terms reflecting preserved interaction, such as alertness, orientation, and verbal responsiveness, were negatively associated with both severity thresholds and were most predictive of mild GCS scores. These findings suggest that respiratory support differentiates mild from nonmild cases, whereas severe GCS is primarily defined by documented neurologic unresponsiveness.

Our error analysis revealed insights into the challenges of GCS prediction. Cases of rapid clinical deterioration, where the documented GCS score failed to capture the patient’s worst neurological state, represented a particular challenge. Similarly, variability in measurement frequency introduced potential bias, as patients with more frequent monitoring may represent a clinically distinct population.

The model’s efficiency is notable—testing on our dataset required only seconds, making it feasible for deployment across large clinical databases. This could enable population-level neurological outcome research that was previously impractical due to the manual effort required for GCS extraction. Applications could include quality improvement initiatives, comparative effectiveness research, and epidemiological studies of neurological conditions. Our approach is extremely fast and scalable compared to large language models, but it is potentially less generalizable and might take longer to develop. State-of-the-art large language models often perform well set to zero-shot, but they are computationally intensive and take time, making them impractical for processing millions of clinical notes in EHR-based research.

### Limitations

Several limitations should be acknowledged. First, both institutions in our study are in the same geographic region, potentially limiting generalizability. We demonstrate that building the model on a single site reduces the performance; therefore, future work should validate the model using data from institutions in different geographies with potentially different documentation practices. Second, our model was developed and validated using retrospective data, and prospective validation would strengthen confidence in its clinical utility. Third, while our model strongly predicted severe GCS scores, further refinement is needed to improve discrimination between moderate and mild categories. With regard to features, our models include vasopressors, which are not direct measures of neurological function and may, in part, act as proxy variables reflecting overall illness severity. However, we chose not to exclude vasopressors because they capture clinically meaningful aspects of a patient’s hemodynamic state, which is often tightly coupled with neurological status in critically ill populations, and removing such variables may therefore omit relevant contextual information. Similarly, while code status terms may introduce proxy-learning behavior, we did not exclude these as they do not inherently equate to greater illness severity but may also reflect patient preferences, comorbidities, or goals of care. Additionally, the feature “unable to assess” may reflect examinations confounded by procedures rather than true neurological impairment; however, this term is also used in clinically unstable patients where a reliable neurological assessment cannot be performed and therefore retained in the model. Another limitation is that our model treats each day independently, ignoring temporal dependencies in GCS trajectories. Future work could use sequence modeling (eg, recurrent neural networks) to better capture patient trajectories. While our model can be applied to notes with fewer than 300 tokens, it cannot be applied in the absence of documentation. Future longitudinal analyses using imputation strategies should include strong clinical caveats, as acute changes in neurological status may not be reliably captured. In addition, aggregating all daily notes into a single representation can dilute short but clinically important events, such as brief deterioration or seizures. This may lead the model to underdetect transient adverse events, representing a structural limitation of the aggregation approach rather than model failure. Future NLP tools should align note time stamps tightly with flowsheet time stamps to resolve this temporal mismatch. Finally, although traditional NLP methods were used in this study, these approaches do not capture contextual or conditional semantics to the same extent as transformer-based models (eg, ClinicalBERT), which may limit sensitivity to complex negations or subtle distinctions within moderate impairment. Future work may evaluate whether contextual embeddings improve performance in these intermediate states.

### Conclusions

Despite the study limitations, our findings demonstrate the feasibility and potential utility of automated GCS prediction from unstructured clinical notes. By enabling efficient, large-scale phenotyping of neurological assessments, this approach could significantly advance population health research and quality improvement efforts in neurological and critical care. Future work should focus on external validation, prospective evaluation, and integration with other clinical decision support tools to maximize impact on patient care.

## Supplementary material

10.2196/81245 Multimedia Appendix 1 Supplemental methods, tables, and figures.

## Abbreviations

AUPRC: area under the precision-recall curve
AUROC: area under the receiver operating characteristic curve
CONSORT-AI: Consolidated Standards of Reporting Trials–Artificial Intelligence
EHR: electronic health record
GCS: Glasgow Coma Scale
MGB: Mass General Brigham
MIMIC: Medical Information Mart for Intensive Care
NLP: natural language processing
RMSE: root mean square error
TRIPOD-AI: Transparent Reporting of a Multivariable Prediction Model for Individual Prognosis or Diagnosis–Artificial Intelligence
