# Supplementary material for: Automated Prediction of Glasgow Coma Scale Scores From Unstructured Electronic Health Records Using Natural Language Processing: Development and Validation Study
Source: J Med Internet Res. 2026 Jun 29;28:e81245. doi: 10.2196/81245 (PMC13313573; doi:10.2196/81245)
Supplement: Multimedia Appendix 1 [file jmir-v28-e81245-s001.docx]

# **Supplemental Material**

## **Supplemental Methods**

### **Notes Preprocessing**

The same notes preprocessing was applied to data from both institutions. Notes were lowercased and text expressions containing special characters were replaced by terms without those characters, such as 'w/out' replaced by 'without'. Text was then processed for removal of special characters and *stop-words*, which consist of frequent and less relevant words. For the primary model in the main manuscript, we removed locations, months, days of the week and alphabet (see Table S1) from the notes. We also conducted a sensitivity analysis with a more extensive list of *stop-words* (Table S1). All models were trained using the identical preprocessing and evaluation framework. Because clinical notes were randomly sampled, this resulted in a slightly different cohort compared to the primary analysis (Table S2, S10). Model performance remained comparable to the primary model (Tables S5-S7, S10-S11) and features importance was similar (Fig. S9). In the sensitivity analysis, the model identified a slightly lower number of top-ranked features compared with the primary pooled ordinal model (445 vs 541). This difference likely reflects the exclusion of common words in the feature set, but the overall predictive performance remained comparable. Model rankings and study conclusions were unchanged.

Numerical values, including directly documented GCS scores, were removed from notes during training and testing of the primary text classification model to prevent data leakage. We evaluated an alternative preprocessing strategy retaining numerical tokens during training; however, inclusion of numerical values resulted in reduced classification performance and was therefore not used in the final model. Duplicated words in a row and duplicated spaces were also removed. Notes were then subjected to *stemming*, i.e. each word was reduced to its root word using the Porter stemming algorithm [1]. We performed abbreviations expansion and contraction to the stemmed words and merged similar words (see Table S1).

Preprocessed notes from each model training set were used to create the training vocabulary. A bag-of-words model was used to represent the notes from each day as a binary vector, indicating the presence of an n-gram (single word or sequence of 2 or 3 words), disregarding grammar and word order, based on the training vocabulary. The number of variables was reduced in the training set at this stage by excluding n-grams that occurred in fewer than 10% of the notes and by removing features with a correlation greater than 95% with other features in the dataset.

We performed a sensitivity analysis to determine the minimum daily note length required to retain sufficient linguistic information for model training. The number and percentage of daily notes excluded and retained according to minimum note-length thresholds are presented in Table S3.

We used the pooled linear model to select an optimal minimum note-length threshold. For each candidate threshold, we trained the pooled linear model and training performance was evaluated at each threshold to identify the optimal minimum note length. We evaluated model performance at thresholds of 150, 200, 250, and 300 words using RMSE and Pearson correlation. Based on this analysis, we selected a 300-word minimum threshold, which achieved the highest correlation (Pearson = 0.81, Table S4) while retaining an adequate number of notes for training. This threshold was applied to the pooled ordinal model to ensure that all included notes contained sufficient information for reliable prediction.

### **Statistical Analysis**

#### Class balance strategy for model training

To address class imbalance and mitigate potential bias towards the majority class during model training, we defined an under-sampling strategy based on the number of samples of the minority GCS class. We selected the minority class from the MGB training set as reference and resampled the model training set by randomly removing samples from the majority classes until they were balanced with this minority class. For under-sampling we used the RandomUnderSampler Python function [2]. This under-sampling strategy was applied to both pooled and single-institution models for linear and ordinal training. For the pooled models, we also applied the Synthetic Minority Over-sampling Technique (SMOTE) [2] to create synthetic samples for minority classes in the MIMIC training set until they were balanced with the MGB minority class. For the ordinal models, we applied the same rebalancing strategies, and selected 15,000 samples per ordinal category per institution to ensure computational feasibility while maintaining the balanced class representation.

#### Ordinal

We developed an ordinal regression model to predict the lowest daily score among severe (GCS 3-8), moderate (GCS 9-12) and mild (GCS 13-15). We fitted a parallel adjacent category probability model with logit link, using the ordinalNet R package [3]. We selected this model as it focuses on comparisons of adjacent categories, comparing a response category to the next response category above it, which is suitable for predicting probabilities on a severity scale. For each daily prediction, we selected the maximum probability out of the three model probabilities. To find the best regularization parameter, we performed five-fold cross-validation within a balanced subsample of 10,000 samples from the training data using the ordinalNetTune R function, with the parameters: “acat” family and “logit” link, standardize, reverse and nonparallelTerms set as TRUE, parallelTerms set as FALSE, nLambda = 3, and remaining default parameters. The algorithm automatically generated a sequence of three values for the regularization parameter. The regularization parameter yielding the minimum error was selected to fit the ordinalNet model in the full training set.

For ordinal performance, confidence intervals (CIs) were estimated using 1,000 bootstrap resamples of fixed size, equal to the smallest test set, drawn with replacement to ensure comparability across datasets.

#### Linear

We developed a linear regression model with the training data using the least absolute shrinkage and selection operator (LASSO) [4] to predict the daily lowest GCS scores 3-15. For this model, we also performed five-fold cross validation within the training data to determine the best regularization parameter. For linear regression, a grid search was performed to select the regularization parameter among the values {0.01, 0.05, 0.1, 0.5, 1}. The regularization parameter yielding the minimum error was selected to fit the LASSO model in the training set. For the linear model, any predicted scores with a negative value below 3 were assigned a value of 3, and above 15 a value of 15. To evaluate the model, we used the root mean squared error (RMSE) and Pearson correlation.

For both ordinal and linear models, age was rescaled and normalized between 0 and 1 using the min-max normalization, based on the reference minimum and maximum age values in the training data. The importance of the variables was assessed by magnitude of the regression coefficients.

To understand variance in results, we performed ten rounds of training undersampling, and for each round we trained and tested a linear regression model in the same training data. Each round was set by changing the random state parameter in the RandomUnderSampler. A random state was randomly selected to under sample both train and test data. This was performed for both ordinal and linear modeling.

## **Supplemental Tables**

**Table S1** List of expressions for each data preprocessing task

| **Task** | **(Expression, replacement)** |
| --- | --- |
| Text processing before special characters or stop words removal | ({'w/out','w out','w/ out'}, 'without')  ({'n’t', 'neither', 'nor'}, 'not')  (' w/', 'with') |
| Stop-words removal | ({#location  'massachusetts', 'ma', 'address', 'newton', 'highland',  'boston', 'fa', 'street','waltham','sommerville',  'avenue','mghw','cambridge','revere','charlestown','wa',  'barrasso','salem','lincoln', 'luckhurst','st','chelsea',  'mghg','nc','mccann','mgh','bwh','webster','lynn','haverhill',  'bi', 'needham', 'massachusetts', 'general', 'hospital',  'pgy2', 'department', 'ellison', 'brigham', 'women',  'logan', 'airport', 'lunder','blake','winthrop','wi','place',  'ave',  #time  'january', 'february', 'march', 'april', 'june', 'july', 'august', 'september', 'october', 'november', 'december', 'monday', 'tuesday', 'wednesday', 'thursday', 'friday', 'saturday', 'sunday'    #alphabet  'a', 'b', 'c', 'd', 'e', 'f', 'g', 'h', 'j', 'k', 'l',  'm', 'n', 'o', 'p', 'q', 'r',  's', 't', 'u', 'v', 'w', 'x', 'y', 'z'}, ' ') |
| Stop-words removal for sensitivity analysis | ({#pronouns  'i', 'me', 'my', 'myself', 'we', 'our', 'ours',  'ourselves', 'you', 'youre', 'youve', 'youll',  'youd', 'your', 'yours', 'yourself', 'yourselves',  'he', 'him', 'his', 'himself', 'she', 'shes',  'her', 'hers', 'herself', 'it', 'its',  'itself', 'they', 'them', 'their', 'theirs',  'themselves',  #verbs  "am", "is", "are", "was", "were", "be", "been", "being",  "have", "has", "had", "having", "do", "does", "did", "doing",  'go', 'went', 'will', "can", 'sent',    #people  'who', "whom", 'mr', 'dr', 'dear', 'resident', 'physician', 'mrn',  'father', 'mother', 'provider', 'ehr', 'rn', 'clinician',  'md', 'inpatient', 'patient', 'sister', 'brother', 'partner', 'ros',  'husband', 'wife', 'spouse', 'person', 'staff', 'name', 'pcp', 'dob',  'medic', 'doct', 'daughter', 'phd', 'pgy', 'family', 'hcp', 'nurse', 'np',  'partners', 'patients', 'pta', 'slp', 'therapist', 'son',  #prepositions etc  'if', 'of', 'ac', 'in', 'out', 'by', 'at', 'fo', 'nan', 'hea', 'pro',  'as', 'or', "a", 'an', 'for', 'the', 'with', 'to', 'be', 'from',  'about', 'should', 'would', 'could', 'same', 'thank', 'please',  'another', 'either', 'every', 'although', 'yet',  'this', 'that', 'what', 'there', 'here', "these", "those", 'also',  'as well', 'too', "because", "until", "while", 'when', "where",  "why", "how", 'then', 'throughout', "against", "between", "into",  "through", "during", "before", "after", "once", "few", "more",  'ever', "on", "off", "over", "under", "again",  "further", 'and', 'which', 'yes', 'other', "most",  "some", "such", "only", "own", "so", "than", "very",  'even', "just", "above", "below",  #words others  'summary', 'facesheet', 'items', 'code', 'phone', 'visit', 'attend',  'encounter', 'note', 'admit', 'admission', 'admitted', 'consult',  'file', 'report', 'page', 'pager', 'comment', 'service',  'relate', 'send', 'edit', 'edited', 'document', 'documentation',  'part', 'use', 'errors', 'set', 'education', 'study', 'find',  'notify', 'systems', 'review', 'assessment', 'pertinent',  'contact', 'outside', 'diagnose', 'information', 'index',  'additional', 'result', 'schedule', 'main', 'former', 'basic',  'comments', 'documented', 'documents', 'data', 'syring', 'test',    #abbr others  'mdd', 'apt', 'resu', 'con', 'dis', 'mch', 'wnl', 'mghe', 'diff',  'id', 'hs', 'hid', 'post', 'nt', 'tid', 'nad', 'pa', 'na', 'msk', 'cc',  're', 'cmf', 'vs',    #location  'massachusetts', 'ma', 'address', 'newton', 'highland',  'boston', 'fa', 'street','waltham','sommerville',  'avenue','mghw','cambridge','revere','charlestown','wa',  'barrasso','salem','lincoln', 'luckhurst','st','chelsea',  'mghg','nc','mccann','mgh','bwh','webster','lynn','haverhill',  'bi', 'needham', 'massachusetts', 'general', 'hospital',  'pgy2', 'department', 'ellison', 'brigham', 'women',  'logan', 'airport', 'lunder','blake','winthrop','wi','place',  'ave',  #time, order, numbers  'date', "first", "second", "third", "fourth", "fifth", "sixth",  "seventh", "eigth", "nineth", "tenth", 'january', 'february',  'march', 'april', 'may', 'june', 'july', 'august', 'september',  'october', 'november', 'december',  'monday', 'tuesday', 'wednesday', 'thursday', 'friday', 'saturday',  'sunday', 'winter', 'spring', 'summer', 'autumn',  'pm', 'yo', 'yr',  #units\|frequency  'mg', 'ml', 'mmhg', 'g', 'cm', 'ii', 'xii', 'lb', 'xl', 'kg',  'pmh', 'unit', 'units', 'qh', 'qd', 'per', 'sig', 'bid',  'mm', 'mcg', 'dl', 'mol', 'mmol', 'neuts', 'oz', 'ng', 'qhs', 'qd', 'pf',    #labs\|vitals  'wbc', 'hgb', 'rdw', 'rbc', 'cbc', 'plt', 'ldl', 'gtt', 'bun',  'hdl', 'glu', 'lipid', 'spo', 'lfts', 'hld', 'hbac', 'hct','ca', 'cr',  'creatinine', 'hb', 'hemoglobin', 'phos', 'potassium', 'magnesium',  'phosphate', 'calcium', 'urea', 'zinc', 'mcv', 'mch', 'mchc',  'sodium', 'chloride', 'ammonia', 'glucose', 'calc',  'monos', 'eos', 'baso', 'basos', 'neutrophils', 'troponin',  'cholesterol', 'triglycerides', 'mpv', 'lymph',  'lymphs', 'mono', 'neutrophil', 'chol', 'nrbc', 'gfr', 'ua',  'cl', 'tp', 'co', 'bun', 'cre', 'pt', 'inr', 'lact', 'tropt',  'sgpt', 'sgot', 'ntbnp', 'alkp', 'tbili', 'dbili', 'alb', 'tsh',  'take', 'tab', 'tablet', 'tablets',  'vitals', 'lab', 'labs',  #alphabet  'a', 'b', 'c', 'd', 'e', 'f', 'g', 'h', 'j', 'k', 'l',  'm', 'n', 'o', 'p', 'q', 'r',  's', 't', 'u', 'v', 'w', 'x', 'y', 'z'}, ' ') |
| Abbreviation expansion | (' hx ', ' histori ')  (' dispo ', ' disposit ')  (' gi ', ' gastrointestin ')  (' gu ', ' genitourinari ')  (' htn ', ' hypertens ')  (' iv ', ' intraven ')  (' dx ', ' diagnosi ')  (' diagnos ', ' diagnosi ')  (' diagnost ', ' diagnosi ')  (' htn ', ' hypertens ')  (' iv ', ' intraven ')  (' pulm ', ' pulmonari ')  (' dm ', ' diabet ')  (' abd ', ' abdomin ')  ('abdomen', 'abdomin')  (' tx ', ' treatment ')  (' etoh ', ' alcohol ')  (' resp ', ' respiratori ')  (' wt ', ' weight ')  (' min ', ' minim ') |
| Abbreviation contraction | ('atrial fibril', 'afib')  ('transient ischaem attack', 'tia')  ('traumatic brain injury', 'tbi')  ('congest heart failur', 'chf')  ('subarachnoid hemorrhag ', 'sah')  ('level conscious', 'loc')  (' short breath ', ' sob ')  ('blood pressur', 'bp') |
| Similar words merge | ({' previous ', ' previou '}, ' previou ')  ({' administr ', ' administ '}, ' administ ')  ({' mildli ', ' mild '}, ' mild ')  ({' surgic ', ' surgeri '}, ' surgeri ')  ({' therapeut ', ' therapi '}, ' therapi ')  ({' anxiou ', ' anxieti '}, ' anxieti ')  ({' bilat ', ' bilater '}, ' bilater ')  ({' infect ', ' infecti '}, ' infecti ') |

**Table S2** Exclusion criteria by institution. The number of patients is represented by ‘n’ and the number of hospital admissions by ‘N’

| **Exclusion criterion** | **MGB (N; n)** | **MIMIC (N; n)** |
| --- | --- | --- |
| Age < 18 years old | 33,826; 24,366 | 8,211; 7,969 |
| Missing total GCS score | 407,412; 209,284 | 1,905; 288 |
| Missing daily notes | 11,855; 4,193 | 768; 568 |
| Missing daily relevant note types | 2,039; 887 | 0; 0 |
| Procedures-only daily notes | 7; 2 | 10,211; 7,511 |
| **Primary analysis** |  |  |
| Note length <300 tokens | 1,352; 766 | 2,072; 1,459 |
| Under-sampling ordinal train set | 424,083; 198,538 | 8,312; 6,085 |
| Under-sampling linear train set | 406,800; 187,277 | 5,941; 4,262 |
| **Sensitivity analysis** |  |  |
| Note length <300 tokens | 2,286; 1,308 | 5,106; 3,554 |
| Under-sampling ordinal train set | 422,549; 198,261 | 6,465; 4,809 |
| Under-sampling linear train set | 404,848; 186,584 | 20,136; 16,689 |

MGB – Massachusetts General Brigham dataset; MIMIC – Medical Information Mart for Intensive Care dataset.

**Table S3** Number and percentage of daily notes excluded and retained according to minimum note-length thresholds

|  | **MIMIC** | | |  | **MGB** | | |
| --- | --- | --- | --- | --- | --- | --- | --- |
| **Threshold** | **#Notes excluded** | **Excluded (%)** | **Retained (%)** |  | **#Notes excluded** | **Excluded (%)** | **Retained (%)** |
| 100 | 6363 | 3.4 | 96.6 |  | 21536 | 0.5 | 99.5 |
| 150 | 15204 | 8.0 | 92.0 |  | 34926 | 0.8 | 99.2 |
| 200 | 26831 | 14.2 | 85.8 |  | 55587 | 1.3 | 98.7 |
| 250 | 40817 | 21.6 | 78.4 |  | 84405 | 1.9 | 98.1 |
| 300 | 56046 | 29.6 | 70.4 |  | 118302 | 2.7 | 97.3 |
| 350 | 70733 | 37.4 | 62.6 |  | 156635 | 3.5 | 96.5 |
| 400 | 84052 | 44.4 | 55.6 |  | 200301 | 4.5 | 95.5 |
| 450 | 95593 | 50.5 | 49.5 |  | 250030 | 5.6 | 94.4 |
| 500 | 105592 | 55.8 | 44.2 |  | 304577 | 6.9 | 93.1 |

#Notes indicates number of notes after applying the minimum tokens threshold. MGB – Massachusetts General Brigham dataset; MIMIC – Medical Information Mart for Intensive Care dataset

**Table S4** Sensitivity analysis of the pooled linear regression model performance evaluated on the training set, showing 95% confidence intervals (CI) for different note length thresholds

| **Threshold** | **RMSE**  **[95% CI]** | **Pearson Correlation**  **[95% CI]** |
| --- | --- | --- |
| 150 | 2.26  [2.26, 2.27] | 0.80  [0.80, 0.80] |
| 200 | 2.25  [2.24, 2.25] | 0.80  [0.80, 0.80] |
| 250 | 2.24  [2.23, 2.25] | 0.80  [0.80, 0.80] |
| 300 | 2.22  [2.21, 2.22] | 0.81  [0.81, 0.81] |

RMSE – root mean square error.

**Table S5** Performance of the pooled linear model before and after calibration on the hold-out test set for the study cohorts, with 95% confidence intervals (CI)

|  | **RMSE before calibration**  **[95% CI]** | **RMSE after calibration**  **[95% CI]** | **Pearson correlation**  **[95% CI]** |
| --- | --- | --- | --- |
| **Primary model** |  |  |  |
| **Pooled** | 2.40  [2.40, 2.41] | 2.30  [2.30, 2.30] | 0.76  [0.76, 0.76] |
| **MGB** | 2.40  [2.40, 2.41] | 2.29  [2.29, 2.30] | 0.74  [0.74, 0.74] |
| **MIMIC** | 2.47  [2.45, 2.48] | 2.43  [2.41, 2.45] | 0.82  [0.82, 0.83] |
| **Sensitivity model** |  |  |  |
| **Pooled** | 2.39  [2.39, 2.40] | 2.29  [2.29, 2.29] | 0.76  [0.75, 0.76] |
| **MGB** | 2.39  [2.39, 2.39] | 2.29  [2.28, 2.29] | 0.74  [0.74, 0.74] |
| **MIMIC** | 2.45  [2.43, 2.47] | 2.42  [2.40, 2.44] | 0.82  [0.82, 0.83] |

MGB – Massachusetts General Brigham dataset; MIMIC – Medical Information Mart for Intensive Care dataset; RMSE – root mean square error.

**Table S6** Performance of the pooled and single-institution ordinal models before calibration for the study cohorts on the hold-out test set, with 95% confidence intervals (CI)

|  | **AUROC**  **[95% CI]** | **Recall**  **[95% CI]** | | **Specificity**  **[95% CI]** | | **F1**  **[95% CI]** | | **AUPRC**  **[95% CI]** | | **PPV**  **[95% CI]** | | **NPV**  **[95% CI]** |
| --- | --- | --- | --- | --- | --- | --- | --- | --- | --- | --- | --- | --- |
| **Primary model** | |  | |  | |  | |  | |  | |  |
| **Pooled model** | |  | |  | |  | |  | |  | |  |
| **Pooled** | 0.96  [0.96-0.96] | 0.80  [0.79-0.81] | | 0.94  [0.94-0.95] | | 0.74  [0.73-0.74] | | 0.77  [0.76-0.78] | | 0.70  [0.69-0.71] | | 0.87  [0.86-0.87] |
| **MGB** | 0.96  [0.96-0.96] | 0.80  [0.79-0.81] | | 0.94  [0.94-0.95] | | 0.73  [0.72-0.74] | | 0.76  [0.75-0.77] | | 0.69  [0.69-0.70] | | 0.86  [0.86-0.86] |
| **MIMIC** | 0.94  [0.94-0.94] | 0.80  [0.80-0.81] | | 0.91  [0.91-0.91] | | 0.80  [0.80-0.81] | | 0.87  [0.86-0.87] | | 0.80  [0.80-0.81] | | 0.91  [0.91-0.91] |
| **Pooled classes** | |  | |  | |  | |  | |  | |  |
| Severe | 0.98  [0.98-0.98] | 0.79  [0.77-0.81] | | 0.98  [0.98-0.98] | | 0.75  [0.74-0.76] | | 0.83  [0.80-0.83] | | 0.72  [0.70-0.73] | | 0.99  [0.99-0.99] |
| Moderate | 0.92  [0.92-0.93] | 0.69  [0.68-0.71] | | 0.92  [0.92-0.92] | | 0.51  [0.49-0.52] | | 0.50  [0.47-0.51] | | 0.41  [0.39-0.41] | | 0.98  [0.97-0.98] |
| Mild | 0.98  [0.97-0.98] | 0.93  [0.92-0.93] | | 0.92  [0.92-0.93] | | 0.96  [0.95-0.96] | | 1.00  [1.00-1.00] | | 0.99  [0.99-0.99] | | 0.65  [0.63-0.65] |
|  |  |  | |  | |  | |  | |  | |  |
| **Single institution model** | | |  | |  | |  | |  | |  | |
| **MIMIC** | 0.90  [0.89-0.90] | 0.73  [0.73-0.74] | | 0.87  [0.87-0.87] | | 0.73  [0.72-0.73] | | 0.80  [0.80-0.81] | | 0.74  [0.74-0.75] | | 0.87  [0.86-0.87] |
| **Classes** |  |  | |  | |  | |  | |  | |  |
| Severe | 0.90  [0.90-0.90] | 0.68  [0.67-0.69] | | 0.92  [0.91-0.92] | | 0.74  [0.73-0.74] | | 0.83  [0.82-0.83] | | 0.80  [0.79-0.80] | | 0.86  [0.85-0.86] |
| Moderate | 0.85  [0.85-0.85] | 0.77  [0.76-0.78] | | 0.76  [0.75-0.76] | | 0.64  [0.63-0.64] | | 0.66  [0.65-0.67] | | 0.54  [0.53-0.55] | | 0.90  [0.90-0.90] |
| Mild | 0.94  [0.94-0.94] | 0.75  [0.74-0.75] | | 0.94  [0.94-0.94] | | 0.81  [0.81-0.82] | | 0.92  [0.92-0.92] | | 0.90  [0.89-0.90] | | 0.84  [0.84-0.85] |
| **Sensitivity model** | |  | |  | |  | |  | |  | |  |
| **Pooled model** | |  | |  | |  | |  | |  | |  |
| **Pooled** | 0.96  [0.96-0.96] | 0.80  [0.79-0.81] | | 0.94  [0.94-0.94] | | 0.74  [0.73-0.74] | | 0.77  [0.77-0.78] | | 0.70  [0.69-0.71] | | 0.87  [0.86-0.87] |
| **MGB** | 0.96  [0.96-0.96] | 0.80  [0.79-0.81] | | 0.94  [0.94-0.94] | | 0.73  [0.72-0.73] | | 0.76  [0.74-0.76] | | 0.69  [0.68-0.69] | | 0.86  [0.86-0.86] |
| **MIMIC** | 0.93  [0.93-0.93] | 0.80  [0.80-0.80] | | 0.91  [0.91-0.91] | | 0.80  [0.80-0.80] | | 0.86  [0.86-0.87] | | 0.80  [0.80-0.80] | | 0.91  [0.91-0.91] |
| **Pooled classes** | |  | |  | |  | |  | |  | |  |
| Severe | 0.98  [0.98-0.99] | 0.78  [0.77-0.81] | | 0.98  [0.98-0.98] | | 0.74  [0.73-0.76] | | 0.82  [0.82-0.84] | | 0.71  [0.69-0.72] | | 0.99  [0.99-0.99] |
| Moderate | 0.92  [0.92-0.93] | 0.69  [0.67-0.70] | | 0.93  [0.92-0.93] | | 0.51  [0.50-0.52] | | 0.50  [0.48-0.52] | | 0.41  [0.39-0.42] | | 0.98  [0.97-0.98] |
| Mild | 0.98  [0.97-0.98] | 0.93  [0.92-0.93] | | 0.92  [0.91-0.93] | | 0.96  [0.95-0.96] | | 1.00  [1.00-1.00] | | 0.99  [0.99-0.99] | | 0.65  [0.63-0.65] |
|  |  |  | |  | |  | |  | |  | |  |
| **Single institution model** | | |  | |  | |  | |  | |  | |
| **MIMIC** | 0.89  [0.89-0.89] | 0.72  [0.72-0.73] | | 0.87  [0.86-0.87] | | 0.72  [0.71-0.72] | | 0.79  [0.79-0.80] | | 0.73  [0.73-0.73] | | 0.86  [0.86-0.86] |
| **Classes** |  |  | |  | |  | |  | |  | |  |
| Severe | 0.89  [0.89-0.90] | 0.74  [0.73-0.75] | | 0.88  [0.87-0.88] | | 0.75  [0.74-0.76] | | 0.83  [0.82-0.84] | | 0.76  [0.75-0.77] | | 0.86  [0.86-0.87] |
| Moderate | 0.84  [0.83-0.84] | 0.71  [0.70-0.72] | | 0.78  [0.77-0.78] | | 0.62  [0.61-0.62] | | 0.65  [0.64-0.66] | | 0.54  [0.53-0.55] | | 0.88  [0.88-0.88] |
| Mild | 0.94  [0.94-0.94] | 0.71  [0.71-0.72] | | 0.95  [0.94-0.95] | | 0.79  [0.79-0.80] | | 0.91  [0.90-0.91] | | 0.89  [0.88-0.90] | | 0.84  [0.84-0.85] |

AUPRC – Area Under the Precision-Recall Curve; AUROC – Area Under the Receiver Operating Characteristic curve; MGB – Massachusetts General Brigham dataset; MIMIC – Medical Information Mart for Intensive Care dataset; PPV – Positive Predictive Value; NPV – Negative Predictive Value.

**Table S7** Performance of the pooled and single-institution ordinal models after calibration for the study cohorts on the hold-out test set, with 95% confidence intervals (CI)

|  | **AUROC**  **[95% CI]** | **Recall**  **[95% CI]** | | **Specificity**  **[95% CI]** | | **F1**  **[95% CI]** | | **AUPRC**  **[95% CI]** | | **PPV**  **[95% CI]** | | **NPV**  **[95% CI]** |
| --- | --- | --- | --- | --- | --- | --- | --- | --- | --- | --- | --- | --- |
| **Primary model** | | |  | |  | |  | |  | |  | |
| **Pooled model** | | |  | |  | |  | |  | |  | |
| **Pooled** | 0.96  [0.96-0.96] | 0.80  [0.79-0.81] | | 0.94  [0.94-0.94] | | 0.73  [0.73-0.74] | | 0.77  [0.76-0.77] | | 0.70  [0.69-0.70] | | 0.86  [0.86-0.86] |
| **MGB** | 0.96  [0.96-0.96] | 0.80  [0.79-0.81] | | 0.94  [0.94-0.95] | | 0.72  [0.72-0.73] | | 0.76  [0.75-0.77] | | 0.69  [0.68-0.70] | | 0.85  [0.85-0.85] |
| **MIMIC** | 0.94  [0.94-0.94] | 0.80  [0.80-0.81] | | 0.91  [0.91-0.91] | | 0.80  [0.80-0.81] | | 0.87  [0.86-0.87] | | 0.80  [0.80-0.81] | | 0.91  [0.91-0.91] |
| **Pooled classes** | | |  | |  | |  | |  | |  | |
| Severe | 0.98  [0.98-0.98] | 0.79  [0.77-0.80] | | 0.98  [0.98-0.98] | | 0.76  [0.74-0.77] | | 0.83  [0.80-0.83] | | 0.73  [0.70-0.74] | | 0.99  [0.99-0.99] |
| Moderate | 0.92  [0.92-0.93] | 0.71  [0.69-0.72] | | 0.91  [0.91-0.91] | | 0.50  [0.48-0.51] | | 0.49  [0.46-0.50] | | 0.39  [0.37-0.39] | | 0.98  [0.97-0.98] |
| Mild | 0.98  [0.97-0.98] | 0.92  [0.91-0.92] | | 0.93  [0.93-0.94] | | 0.95  [0.95-0.95] | | 1.00  [1.00-1.00] | | 0.99  [0.99-0.99] | | 0.62  [0.61-0.63] |
| **Single institution model** | | |  | |  | |  | |  | |  | |
| **MIMIC** | 0.90  [0.89-0.90] | 0.72  [0.71-0.72] | | 0.87  [0.86-0.87] | | 0.71  [0.71-0.72] | | 0.80  [0.79-0.80] | | 0.74  [0.74-0.75] | | 0.86  [0.86-0.86] |
| **Classes** | | |  | |  | |  | |  | |  | |
| Severe | 0.90  [0.90-0.90] | 0.67  [0.66-0.67] | | 0.92  [0.92-0.93] | | 0.73  [0.72-0.74] | | 0.83  [0.82-0.83] | | 0.81  [0.80-0.81] | | 0.85  [0.85-0.86] |
| Moderate | 0.84  [0.84-0.84] | 0.80  [0.78-0.80] | | 0.72  [0.71-0.72] | | 0.62  [0.61-0.62] | | 0.65  [0.64-0.66] | | 0.51  [0.50-0.51] | | 0.91  [0.90-0.91] |
| Mild | 0.95  [0.94-0.95] | 0.70  [0.69-0.71] | | 0.96  [0.95-0.96] | | 0.79  [0.79-0.80] | | 0.92  [0.92-0.92] | | 0.92  [0.91-0.92] | | 0.82  [0.82-0.83] |
| **Sensitivity model** | | |  | |  | |  | |  | |  | |
| **Pooled model** | | |  | |  | |  | |  | |  | |
| **Pooled** | 0.96  [0.96-0.96] | 0.80  [0.79-0.81] | | 0.94  [0.93-0.94] | | 0.73  [0.73-0.74] | | 0.77  [0.76-0.78] | | 0.70  [0.69-0.70] | | 0.86  [0.86-0.86] |
| **MGB** | 0.96  [0.96-0.96] | 0.80  [0.79-0.81] | | 0.94  [0.94-0.94] | | 0.72  [0.71-0.73] | | 0.76  [0.74-0.76] | | 0.68  [0.67-0.69] | | 0.85  [0.85-0.85] |
| **MIMIC** | 0.93  [0.93-0.94] | 0.80  [0.80-0.80] | | 0.91  [0.91-0.91] | | 0.80  [0.80-0.80] | | 0.86  [0.86-0.87] | | 0.80  [0.80-0.80] | | 0.91  [0.90-0.91] |
| **Pooled classes** | | |  | |  | |  | |  | |  | |
| Severe | 0.98  [0.98-0.99] | 0.78  [0.77-0.80] | | 0.98  [0.98-0.98] | | 0.75  [0.74-0.76] | | 0.81  [0.81-0.84] | | 0.72  [0.70-0.73] | | 0.99  [0.99-0.99] |
| Moderate | 0.93  [0.92-0.93] | 0.71  [0.69-0.72] | | 0.92  [0.91-0.92] | | 0.50  [0.48-0.51] | | 0.49  [0.47-0.51] | | 0.38  [0.37-0.40] | | 0.98  [0.98-0.98] |
| Mild | 0.98  [0.97-0.98] | 0.92  [0.91-0.92] | | 0.93  [0.92-0.94] | | 0.95  [0.95-0.95] | | 1.00  [1.00-1.00] | | 0.99  [0.99-0.99] | | 0.62  [0.61-0.63] |
| **Single institution model** | | |  | |  | |  | |  | |  | |
| **MIMIC** | 0.88  [0.88-0.89] | 0.71  [0.70-0.71] | | 0.86  [0.86-0.86] | | 0.70  [0.70-0.71] | | 0.79  [0.79-0.80] | | 0.73  [0.72-0.73] | | 0.85  [0.85-0.86] |
| **Classes** | | |  | |  | |  | |  | |  | |
| Severe | 0.89  [0.89-0.90] | 0.73  [0.71-0.73] | | 0.88  [0.88-0.89] | | 0.75  [0.74-0.75] | | 0.83  [0.82-0.84] | | 0.77  [0.77-0.78] | | 0.86  [0.85-0.86] |
| Moderate | 0.82  [0.82-0.83] | 0.74  [0.73-0.74] | | 0.73  [0.73-0.74] | | 0.60  [0.59-0.60] | | 0.63  [0.62-0.64] | | 0.50  [0.49-0.51] | | 0.88  [0.88-0.89] |
| Mild | 0.94  [0.94-0.94] | 0.66  [0.65-0.67] | | 0.96  [0.96-0.96] | | 0.76  [0.76-0.77] | | 0.91  [0.90-0.91] | | 0.91  [0.91-0.92] | | 0.82  [0.82-0.83] |

AUPRC – Area Under the Precision-Recall Curve; AUROC – Area Under the Receiver Operating Characteristic curve; MGB – Massachusetts General Brigham dataset; MIMIC – Medical Information Mart for Intensive Care dataset; PPV – Positive Predictive Value; NPV – Negative Predictive Value.

**Table S8** Modeling parameters for prediction of the Glasgow Coma Scale (GCS) scores

|  | **Number of selected**  **variables** | **Number of uni, bi, tri-grams** | **Regularization parameter** |
| --- | --- | --- | --- |
| **Primary model** |  |  |  |
| Pooled ordinal | 541 | 401, 125, 15 | 0.001 |
| Single-institution  ordinal | 688 | 458, 195, 35 | 0.001 |
| Pooled linear | 534 | 398, 117, 19 | 0.010 |
| **Sensitivity model** | |  |  |
| Pooled ordinal | 445 | 384, 54, 7 | 0.002 |
| Single-institution  ordinal | 667 | 621, 39, 7 | 0.001 |
| Pooled linear | 489 | 394, 83, 12 | 0.010 |

Ordinal scores: 3-8, 9-12, 13-15. Linear scores: 3-15.

**Table S9** Brier scores of the pooled ordinal model for the study cohorts on the hold-out test set, with 95% confidence intervals (CI)

|  | **Before calibration** | | **After calibration** | |
| --- | --- | --- | --- | --- |
| **Test sets** | **Multiclass Brier**  **[95% CI]** | **Ordinal**  **Brier**  **[95% CI]** | **Multiclass Brier**  **[95% CI]** | **Ordinal**  **Brier**  **[95% CI]** |
| **Primary model** |  |  |  |  |
| **Pooled model** |  |  |  |  |
| **Pooled** | 0.16  [0.16-0.16] | 0.10  [0.09-0.10] | 0.16  [0.16-0.16] | 0.10  [0.09-0.10] |
| **MGB** | 0.15  [0.15-0.16] | 0.09  [0.09-0.10] | 0.16  [0.15-0.16] | 0.09  [0.09-0.10] |
| **MIMIC** | 0.26  [0.26-0.26] | 0.15  [0.14-0.15] | 0.26  [0.26-0.26] | 0.14  [0.14-0.15] |
| **Single-institution** | 0.39  [0.38-0.39] | 0.23  [0.23-0.24] | 0.39  [0.39-0.40] | 0.24  [0.23-0.24] |
| **Sensitivity model** |  |  |  |  |
| **Pooled model** |  |  |  |  |
| **Pooled** | 0.16  [0.15-0.16] | 0.09  [0.09-0.10] | 0.16  [0.15-0.16] | 0.09  [0.09-0.10] |
| **MGB** | 0.15  [0.15-0.16] | 0.09  [0.09-0.09] | 0.15  [0.15-0.16] | 0.09  [0.09-0.09] |
| **MIMIC** | 0.27  [0.27-0.28] | 0.15  [0.15-0.15] | 0.27  [0.27-0.28] | 0.15  [0.15-0.15] |
| **Single-institution** | 0.40  [0.39-0.40] | 0.24  [0.24-0.25] | 0.41  [0.40-0.41] | 0.25  [0.24-0.25] |

MGB – Massachusetts General Brigham dataset; MIMIC – Medical Information Mart for Intensive Care dataset.

**Table S10 Characteristics of the study population for the sensitivity analysis cohort**

| **Characteristic** | **Train set** | | **Test set** | | **Train set**  **(n=40,448)** | **Test set**  **(n=104,365)** |
| --- | --- | --- | --- | --- | --- | --- |
|  | **MGB**  **(n=26,616)** | **MIMIC (n=13,832)** | **MGB (n=96,376)** | **MIMIC (n=7,989)** |  |  |
| **Age (years), mean (SD)** | 64 (17) | 64 (17) | 62 (18) | 63 (18) | 64 (17) | 62 (18) |
| **Male, n (%)** | 14,707 (55) | 7,874 (57) | 48,741 (51) | 4,486 (56) | 22,581 (56) | 53,227 (51) |
| **Race, n (%)** |  |  |  |  |  |  |
| White | 20,572 (81) | 10,056 (73) | 77,128 (80) | 5,829 (73) | 30,628 (76) | 82, 957 (80) |
| Black or African American | 2,372 (9) | 1,014 (7) | 6,912 (7) | 622 (8) | 3,386 (8) | 7,534 (7) |
| Asian | 894 (4) | 322 (2) | 3,101 (3) | 162 (2) | 1,216 (3) | 3,263 (3) |
| Other ^(1)^ | 1,618 (6) | 2,440 (18) | 9,235 (10) | 1,376 (17) | 5,218 (13) | 10,611 (10) |
| **Hispanic or Latino**, **n (%)** | 2,098 (8) | 460 (3) | 7,785 (8) | 226 (3) | 2,558 (6) | 8,011 (8) |
| **Hospital daily stays, D** | 45,000 | 45,000 | 1,301,242 | 40,832 | 90,000 | 1,342,074 |
| **Same day death, D (%)** | 747 (2) | 1,072 (2) | 4,345 (0.3) | 956 (2) | 1,819 (2) | 5,301 (0.4) |
| **Admission type, D (%)** | |  |  |  |  |  |
| Emergency | 28,757 (64) | 38,604 (86) | 866,014 (67) | 35,494 (87) | 67,361 (75) | 901,508 (67) |
| Urgent | 9,214 (20) | 1,677 (4) | 203,216 (16) | 1,310 (3) | 10,891 (12) | 214,107 (16) |
| Elective | 8,100 (18) | 4,719 (10) | 246,013 (19) | 4,029 (10) | 12,819 (14) | 250,042 (19) |
| **GCS classes, D (%)** | |  |  |  |  |  |
| Severe  (3-8) | 15,000 (33) | 15,000 (33) | 62,527 (5) | 14,111 (34) | 30,000 (33) | 76,638 (6) |
| Moderate  (9-12) | 15,000 (33) | 15,000 (33) | 82,182 (6) | 10,908 (27) | 30,000 (33) | 93,090 (7) |
| Mild  (13-15) | 15,000 (33) | 15,000 (33) | 1,156,533 (89) | 15,813 (39) | 30,000 (33) | 1,172,346 (87) |

The number of patients is represented by n and the number of daily inpatient stay is represented by D. ^(1)^ Includes American Indian or Alaska Native, multi-race and unknown race. GCS – Glasgow Coma Scale; Same-day death – death on the same day of inpatient day; SD – standard deviation. Race, ethnicity and same day death are presented for descriptive purposes to characterize the cohort; these variables were not used as predictors in the modeling analyses.

## **Supplemental Figures**

**Fig. S1** Areas under the (a) receiver operating characteristic curve (AUROC) and (b) precision-recall curve (AUPRC), for the primary pooled ordinal model and (c) AUROC and (d) AUPRC for the sensitivity pooled ordinal model, after calibration, evaluated on the full hold-out test set


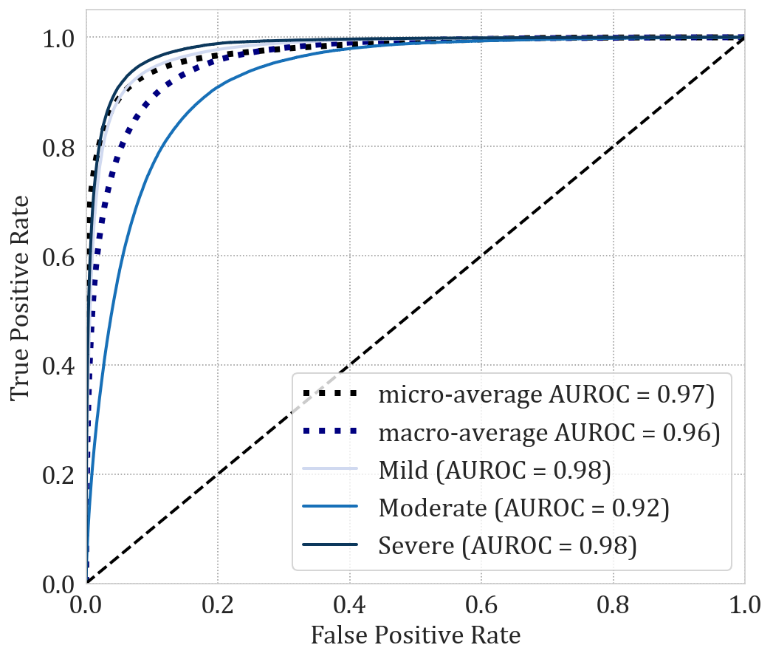


(a)


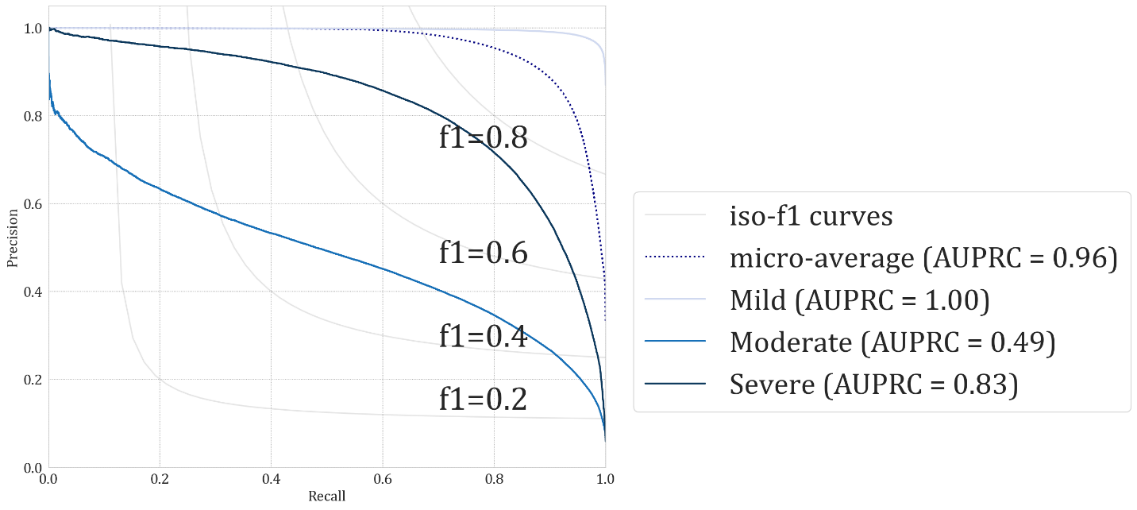


(b)


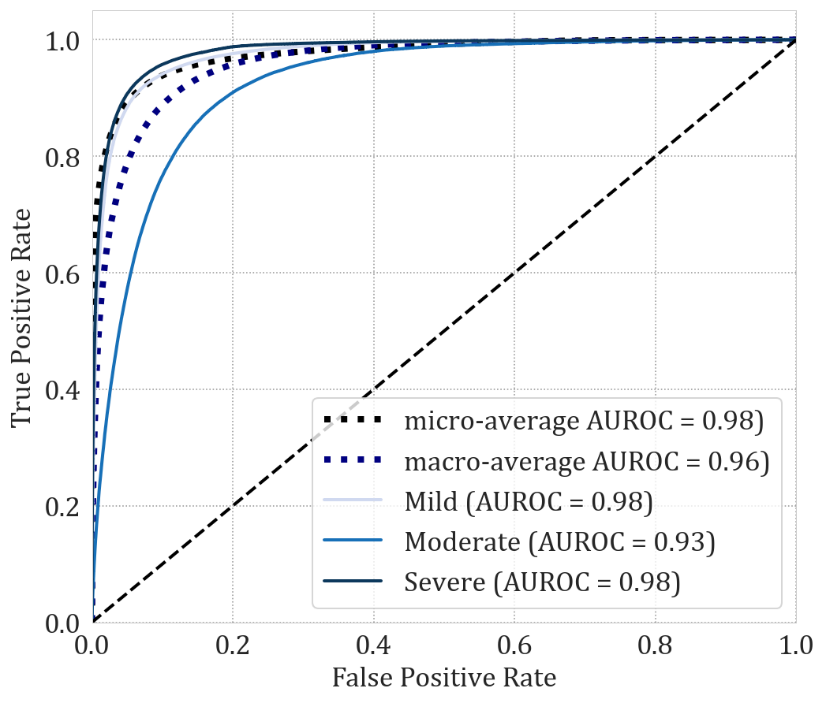


(c)


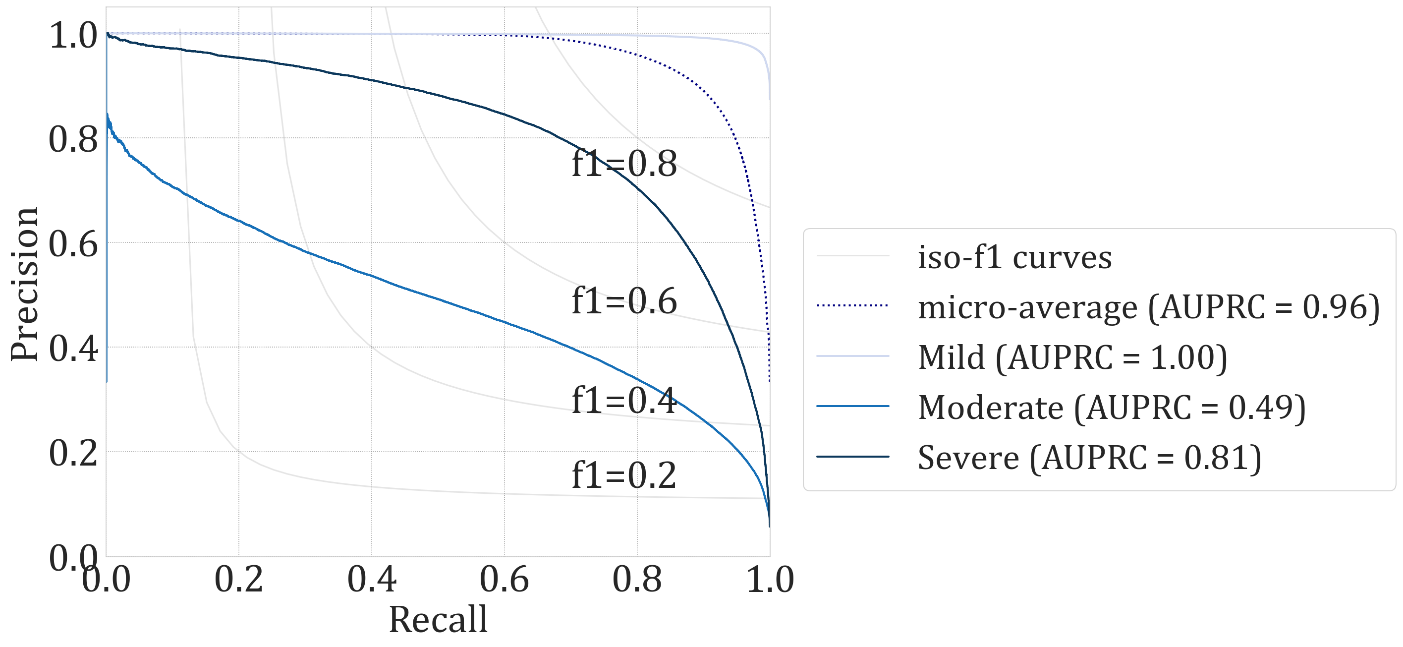


(d)

**Fig. S2** Areas under the (a) receiver operating characteristic curve (AUROC) and (b) precision-recall curve (AUPRC), for the primary pooled ordinal model and (c) AUROC and (d) AUPRC for the sensitivity pooled ordinal model, after calibration, evaluated on the MGB hold-out test set


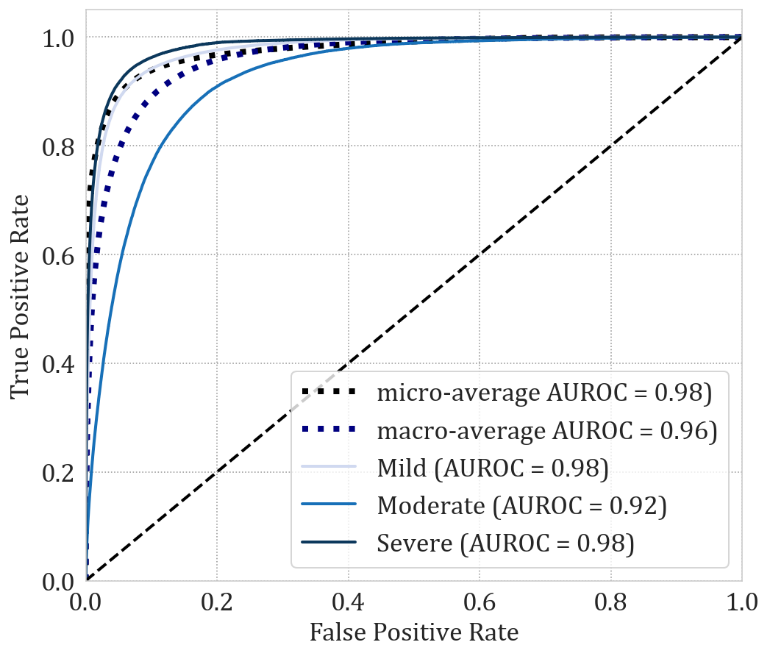


(a)


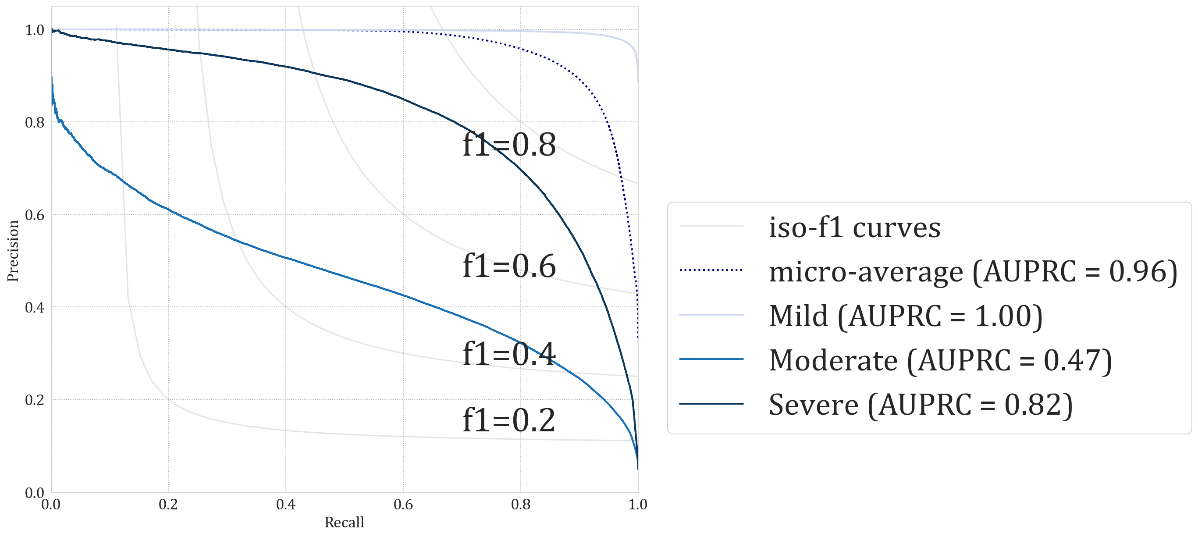


(b)

**
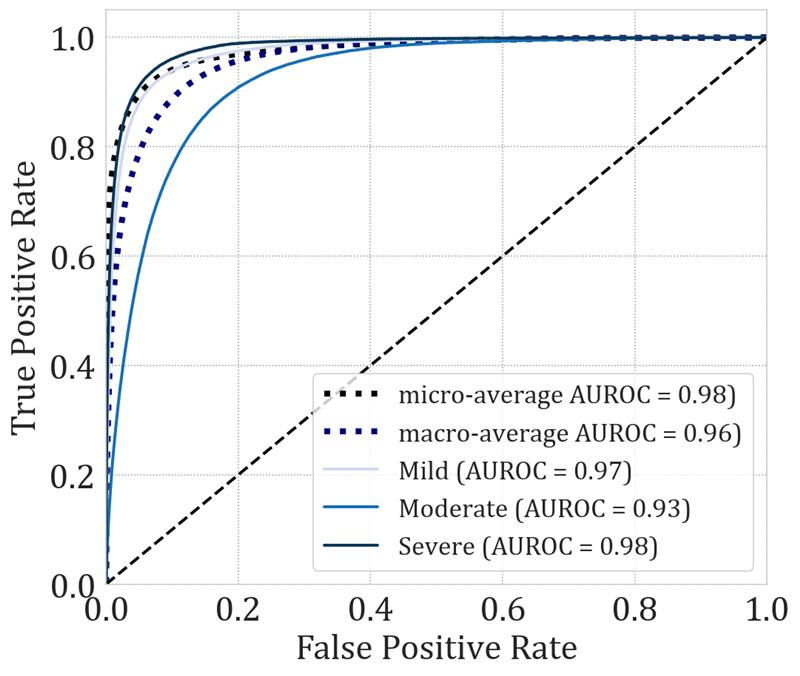
**

(c)

**
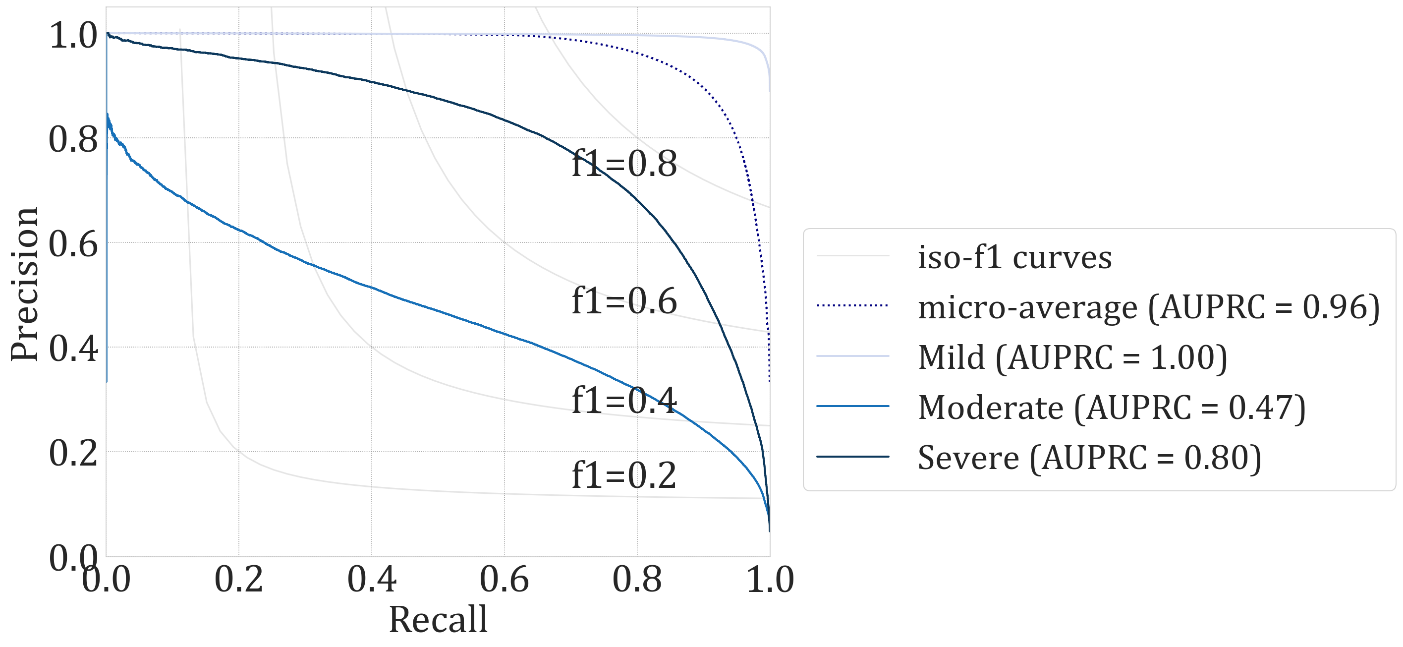
**

(d)

**Fig. S3** Areas under the (a) receiver operating characteristic curve (AUROC) and (b) precision-recall curve (AUPRC), for the primary pooled ordinal model and (c) AUROC and (d) AUPRC for the sensitivity pooled ordinal model, after calibration evaluated on the MIMIC hold-out test set


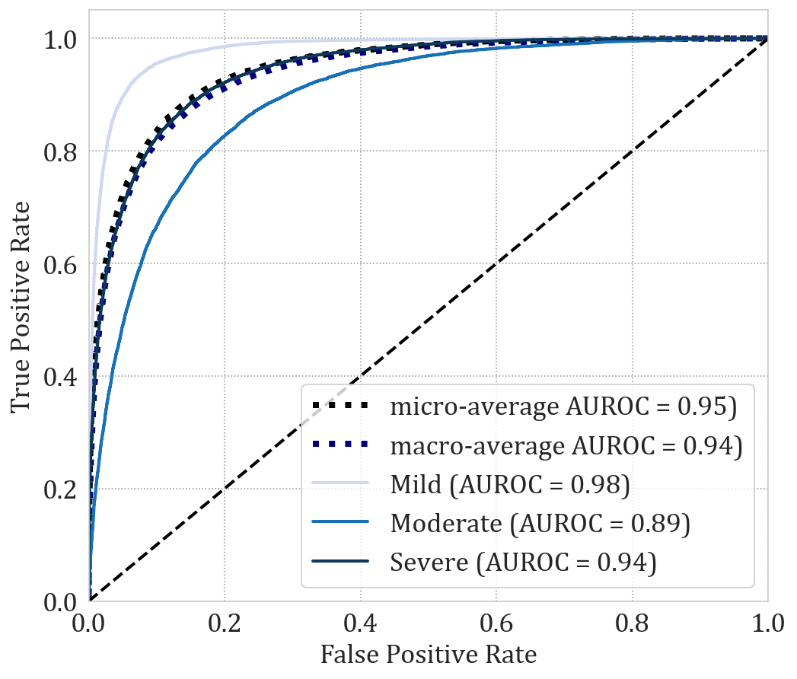


(a)


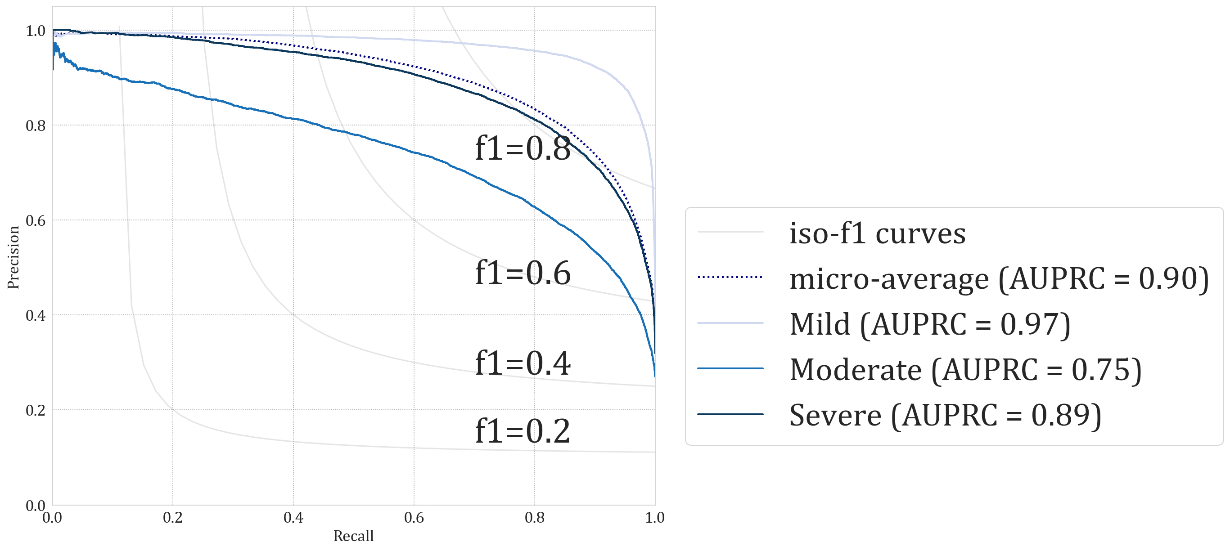


(b)

**
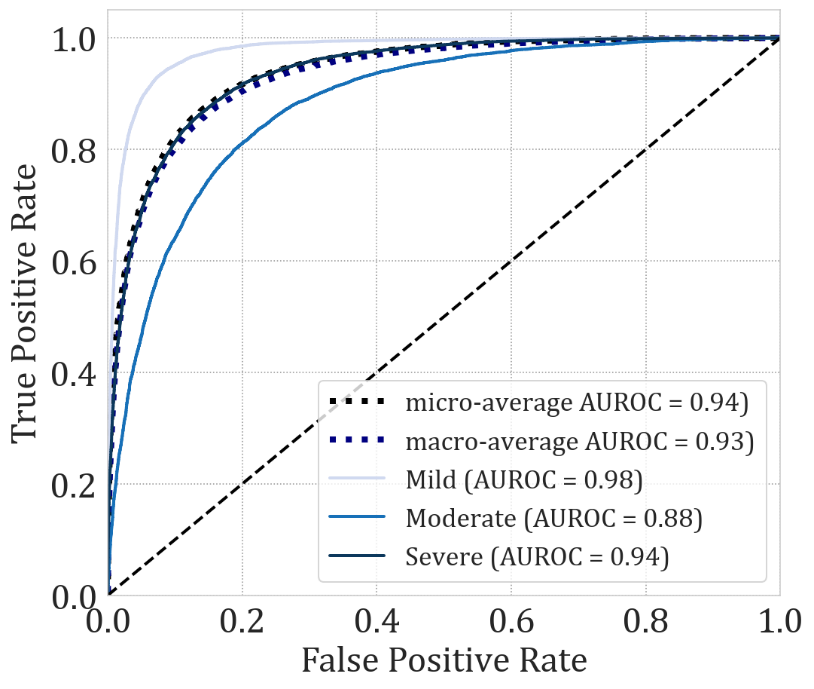
**

(c)

**
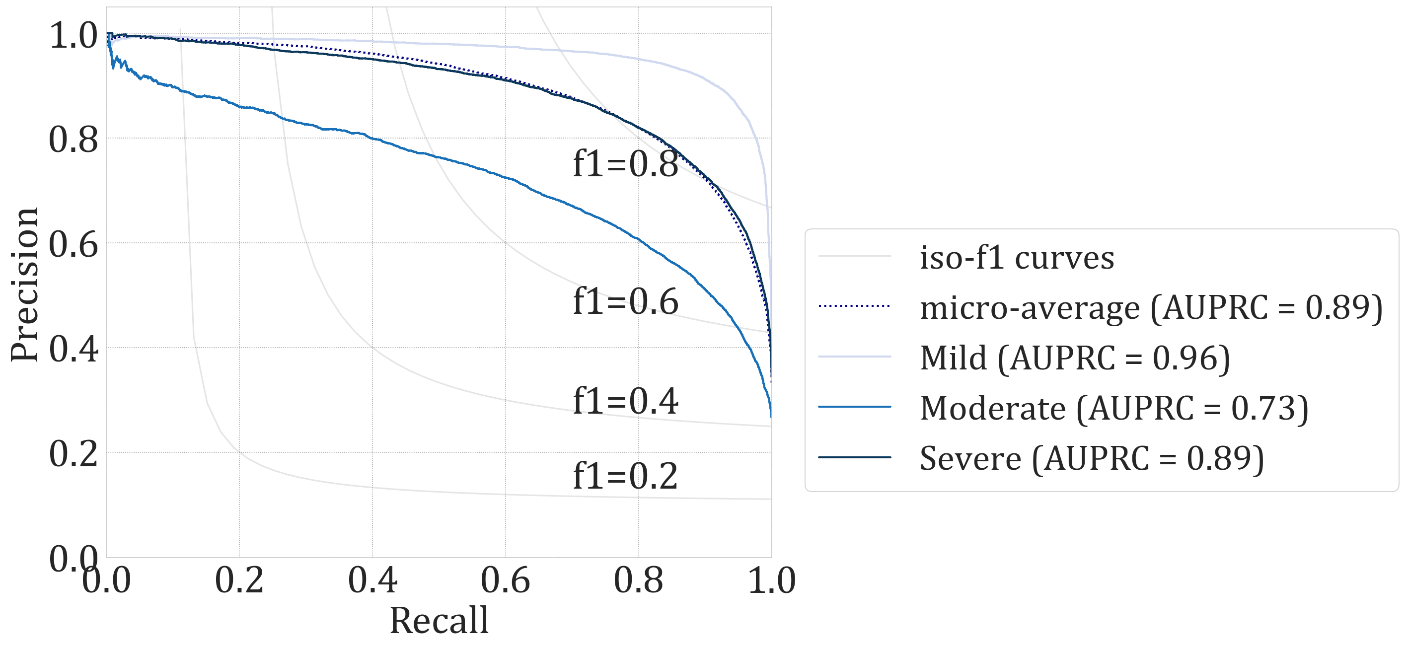
**

(d)

**Fig. S4** Performance of calibrated single-institution ordinal models in the hold-out test set: (a) receiver operating characteristic curve (AUROC), (b) precision-recall curve (AUPRC), and (c) confusion matrix for the primary model, and (d) AUROC, (e) AUPRC, and (f) confusion matrix for the sensitivity model.

**
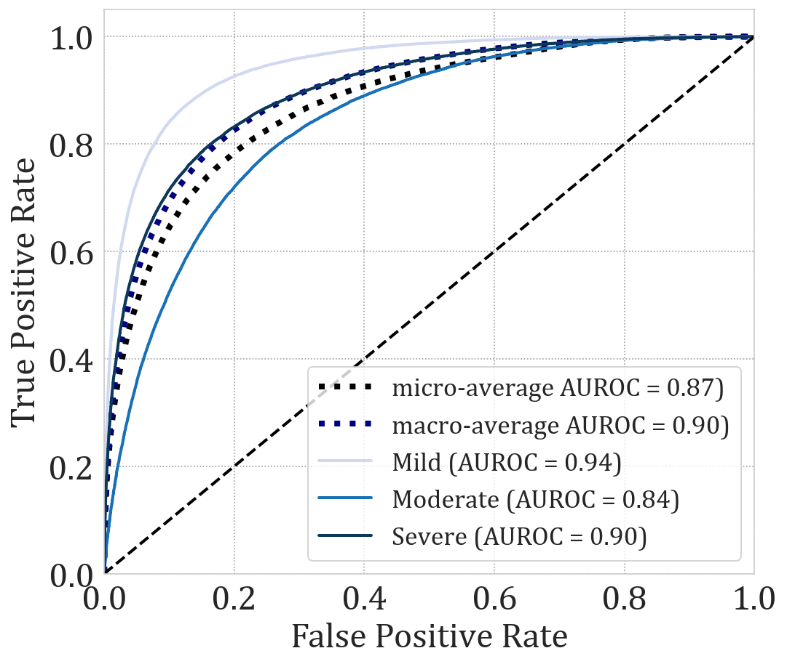
**

(a)


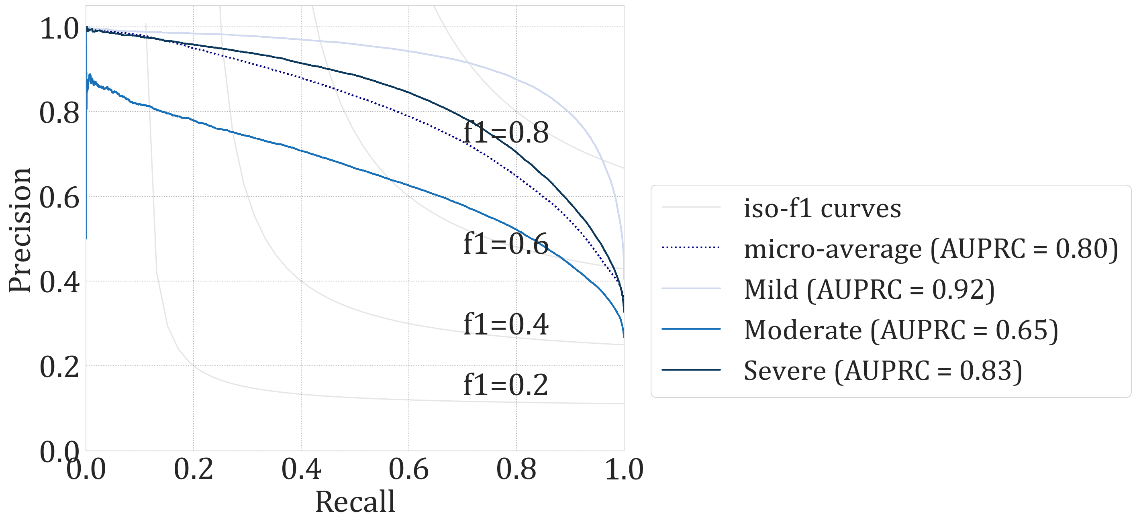


(b)


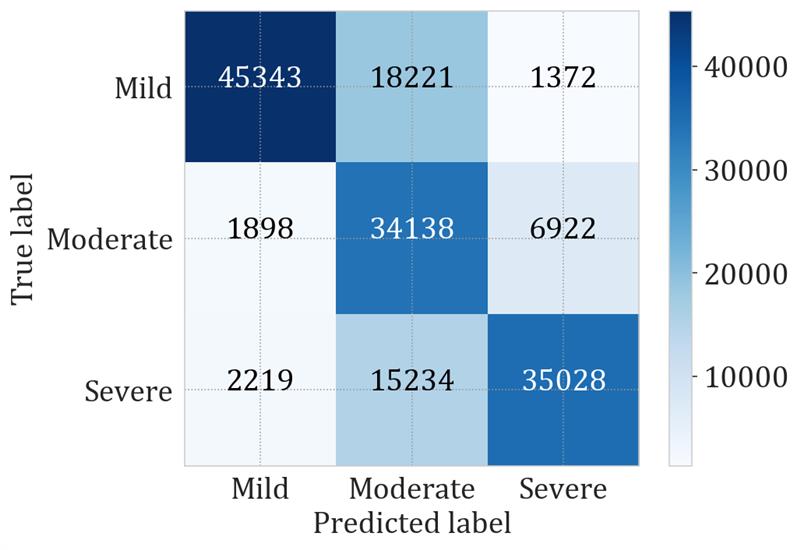


(c)


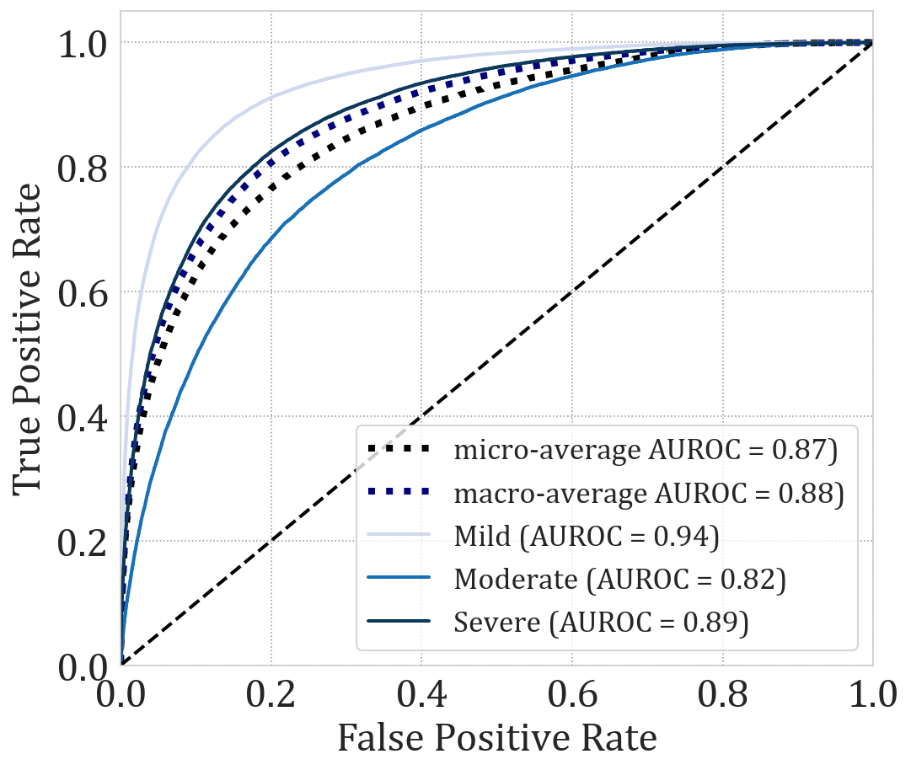


(d)


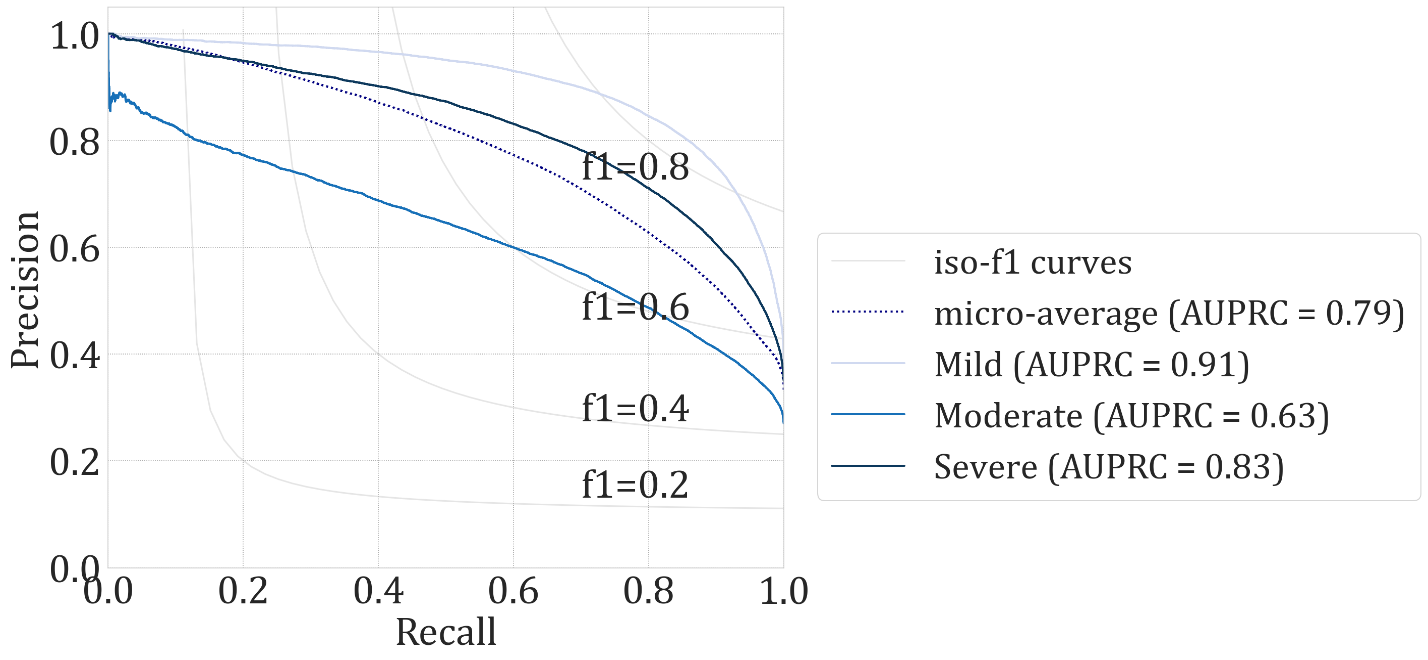


(e)

**
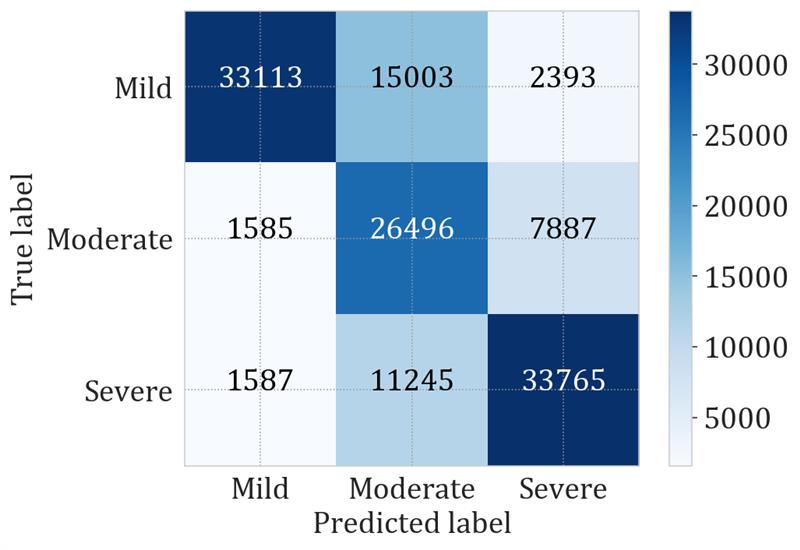
**

(f)

**Fig. S5** CONSORT-AI (Consolidated Standards of Reporting Trials – Artificial Intelligence) charts for modeling: (a) primary linear model, (b) sensitivity linear model and (c) sensitivity ordinal model. The number of patients is represented by ‘n’, the number of hospital admissions by ‘N’ and the number of inpatient days by ‘D’


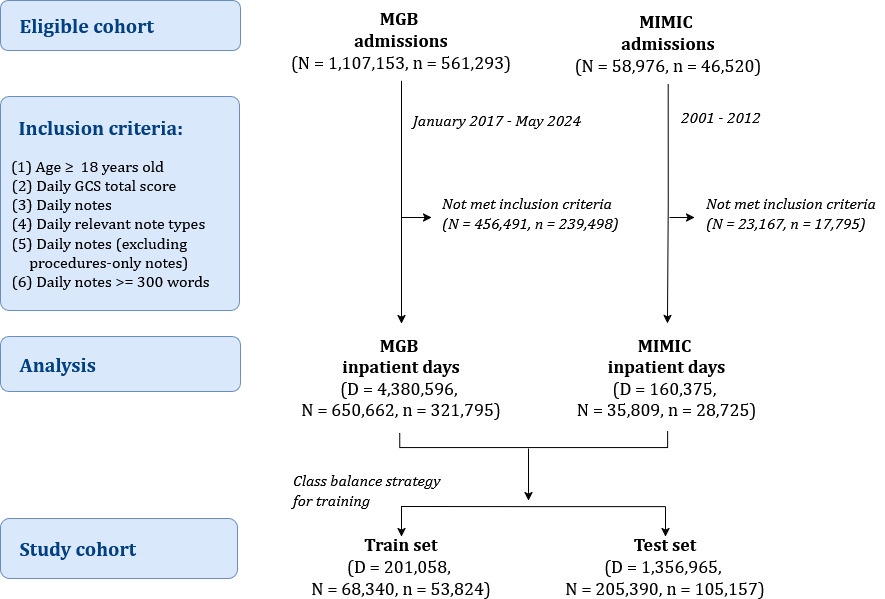


(a)


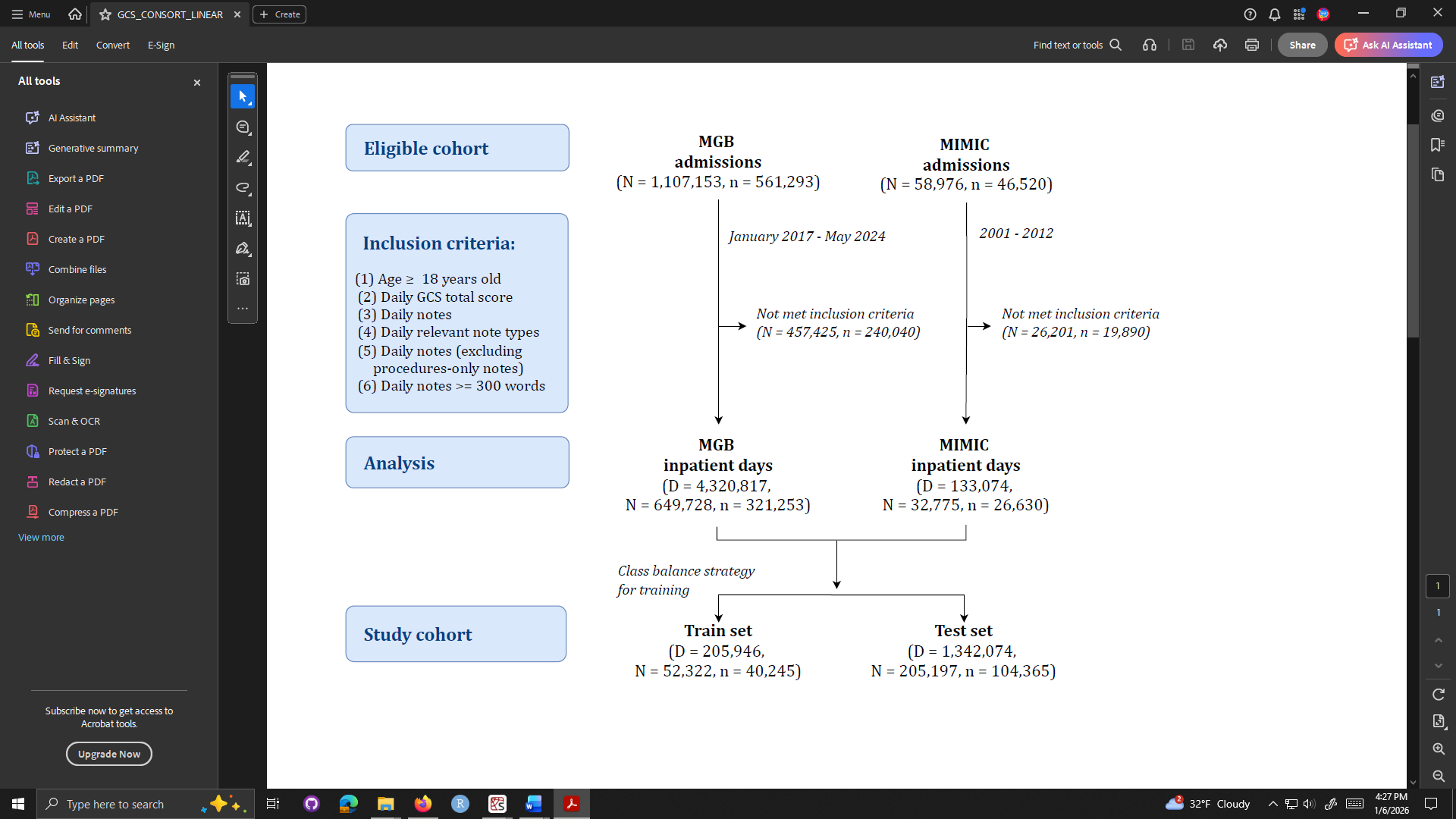


(b)


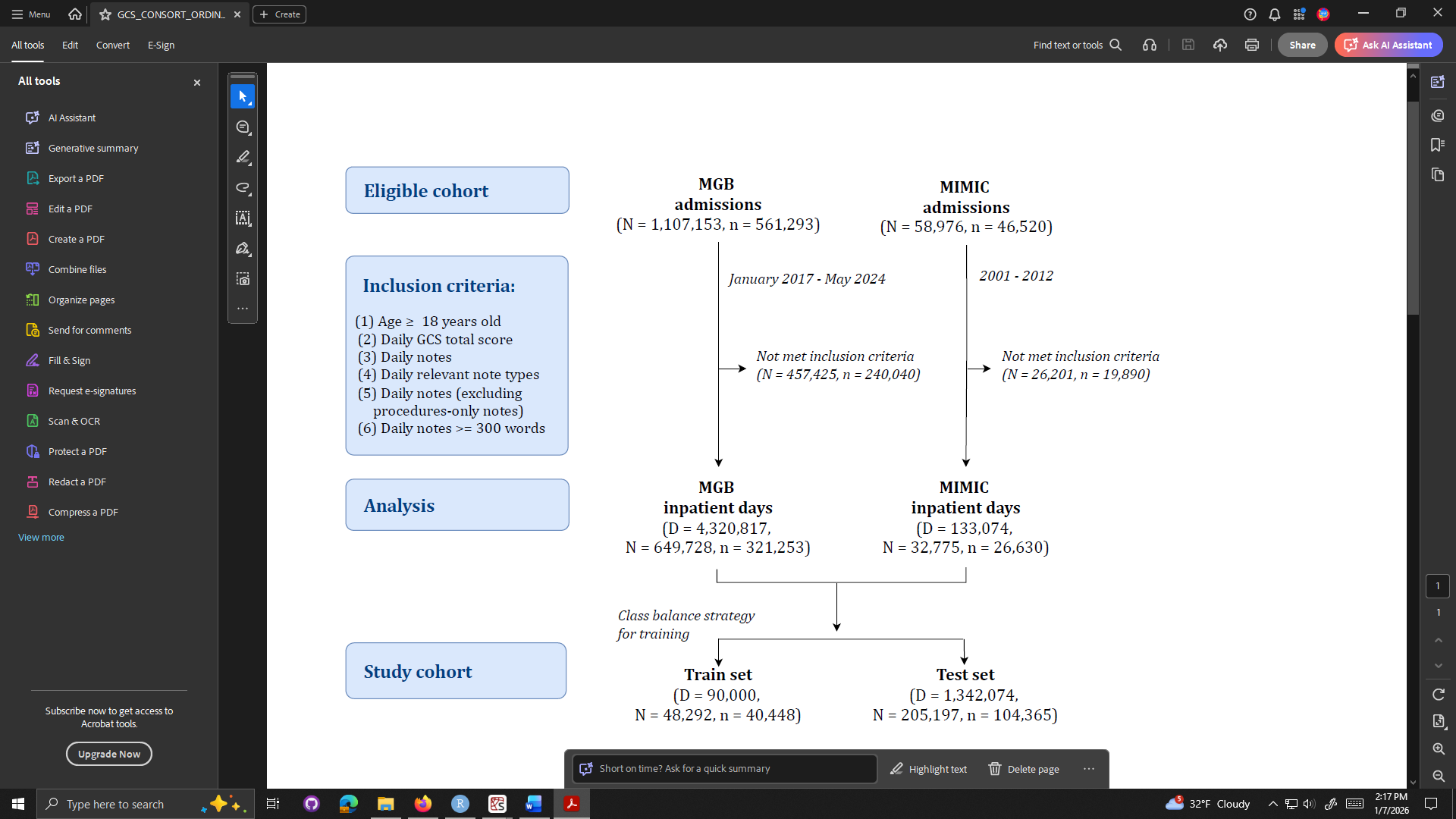


(c)

GCS – Glasgow Coma Scale; MGB - Mass General Brigham; MIMIC – Medical Information Mart for Intensive Care-MIMIC III.

**Fig. S6** Performance of the pooled linear models in the hold-out test set: (a) primary model and (b) sensitivity model, showing predicted vs target GCS scores


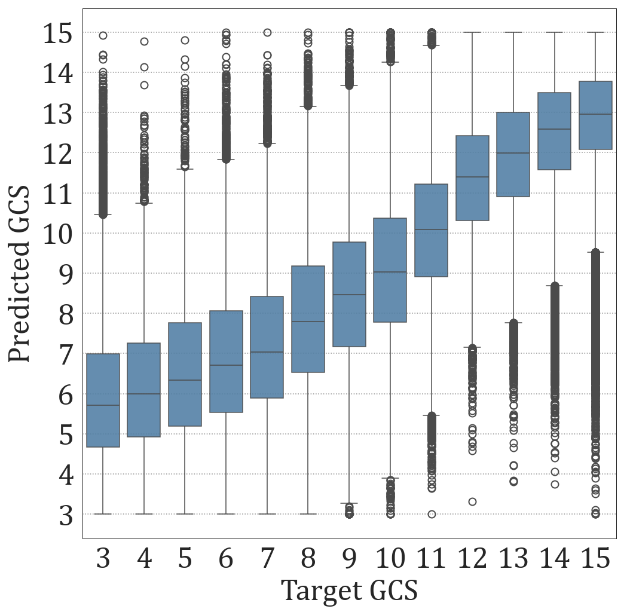

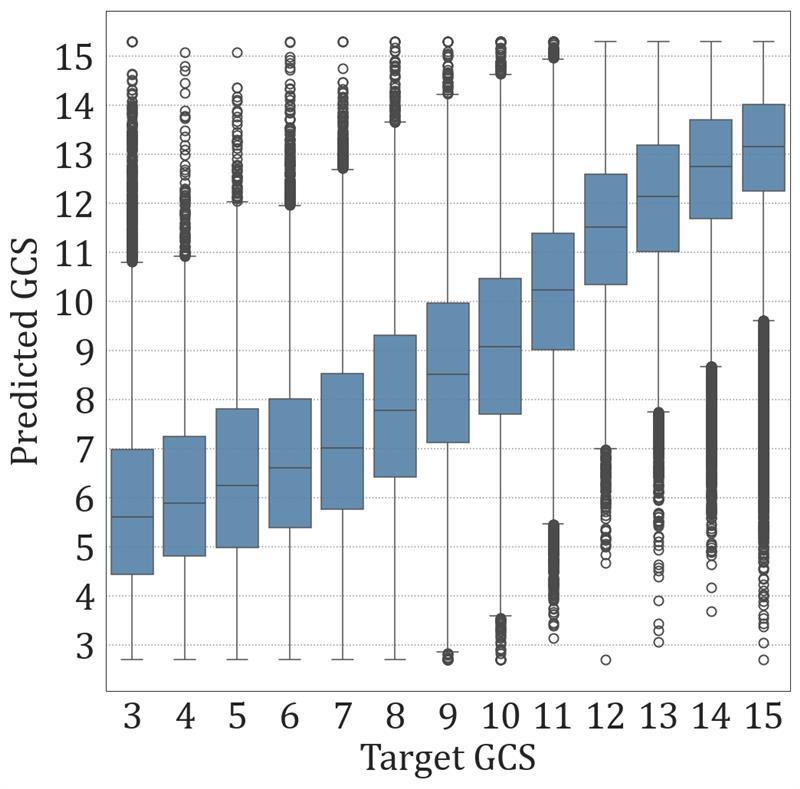


(a) (b)

**Fig. S7** Calibration curve of the pooled linear models in the hold-out test set: (a) primary model and (b) sensitivity model, showing mean predicted vs target GCS scores


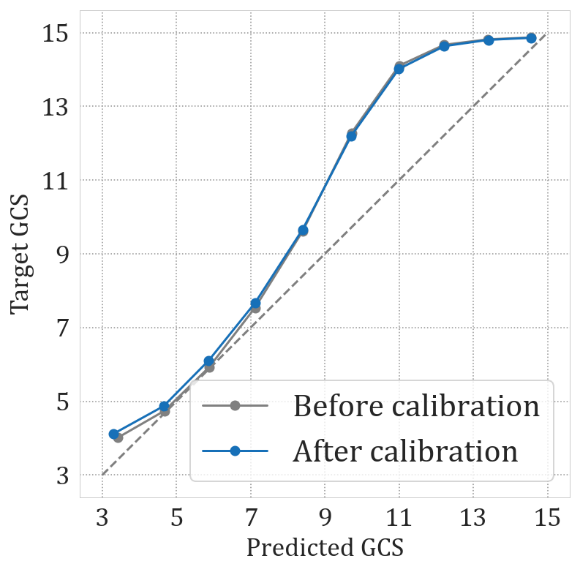

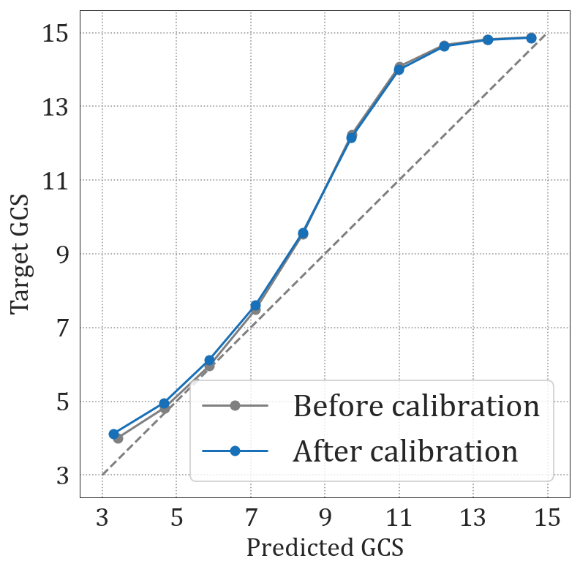


1. (b)

**Fig. S8** Feature importance given by the pooled linear models: (a) primary model and (b) sensitivity model coefficients for the top 20 features. Positive coefficients contribute to higher Glasgow Coma Scale scores while negative coefficients contribute to lower scores


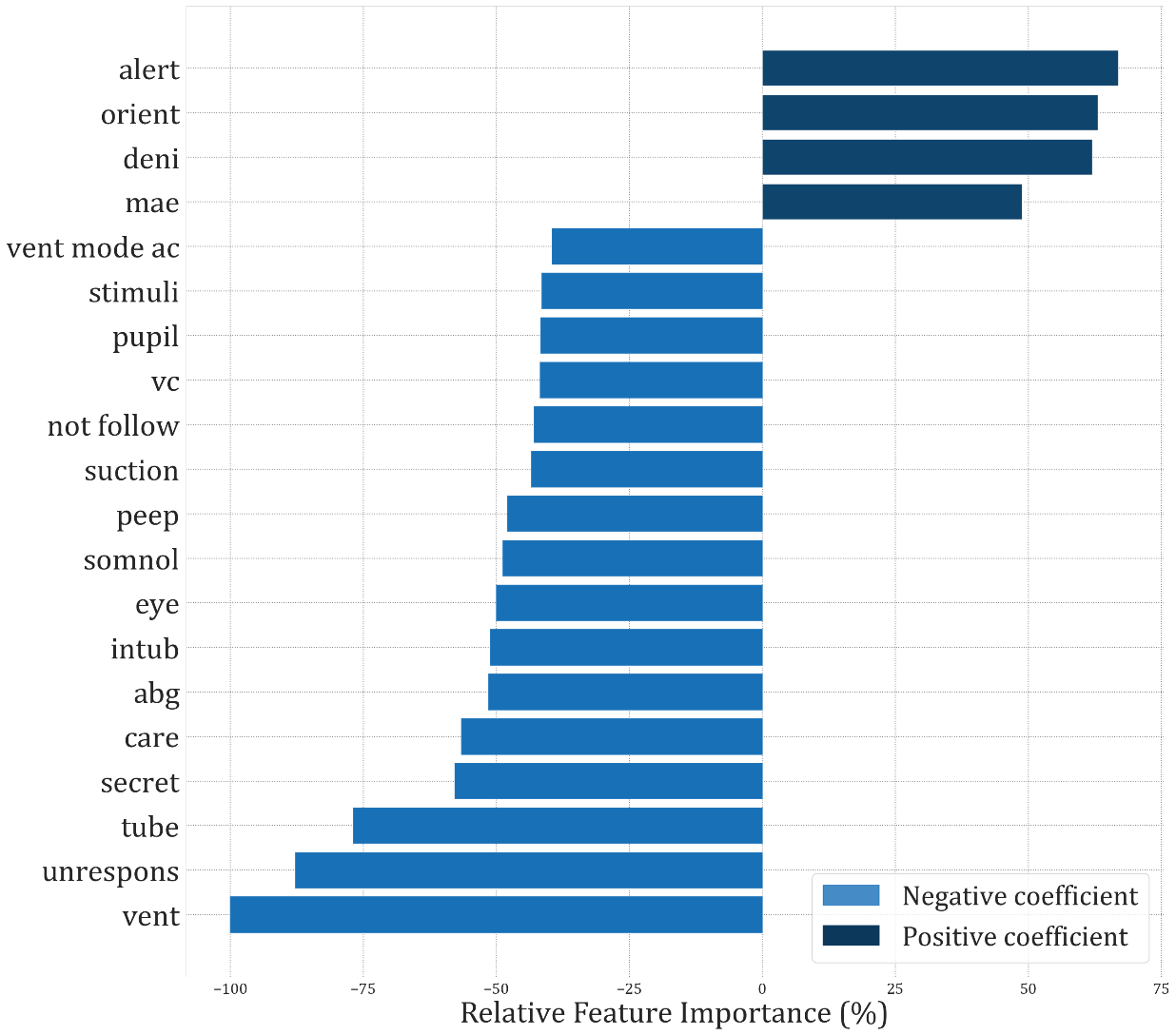


(a)


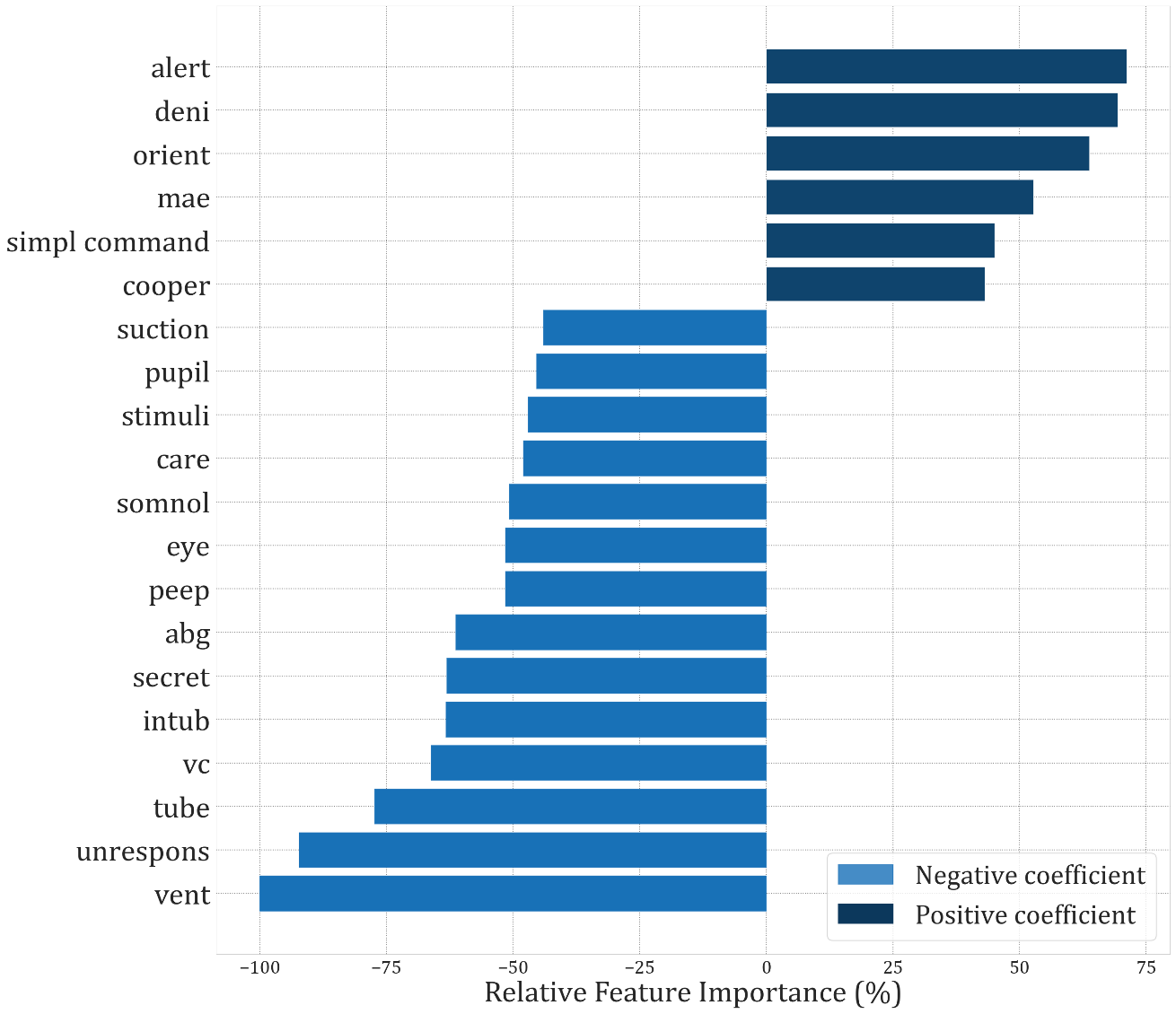


(b)

Legend: Stemmed variables: ‘unrespons’ – unresponsive, ‘vent’ – ventilation, ‘secret’ – secretion (eg. secretion management in mechanical ventilation or intubated patients), ‘intub’ – intubation, ‘somnol’ – somnolence, ‘deni’ – patient denies, ‘orient’ – patient is oriented, ‘simpl command’ – patient follows simple commands, ‘cooper’ – patient is cooperative. Stemmed acronyms: ‘abg’ – arterial blood gases, ‘mae’ – moves all extremities, ‘peep’ – positive end-expiratory pressure, ‘vc’ – vital capacity (respiratory measurement) or volume-control ventilation mode, both indicate respiratory/ventilation status.

**Fig. S9** Top 20 feature importance given by coefficients of the pooled ordinal sensitivity model (a) Threshold 1: Mild vs Moderate + Severe, (b) Threshold 2: Mild + Moderate vs Severe. Positive coefficients indicate higher likelihood of being above the threshold (more severe), while negative coefficients indicate higher likelihood of being below the threshold (less severe)

**
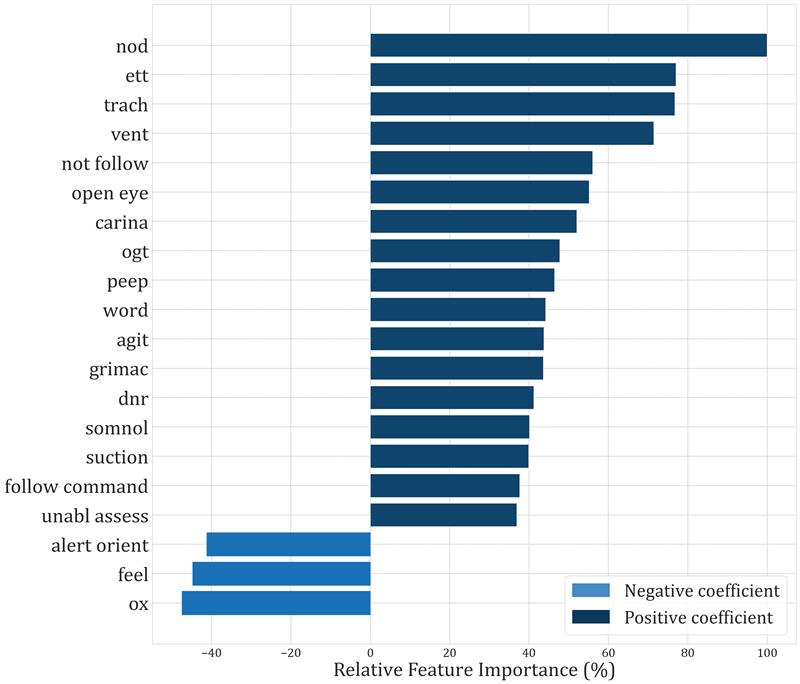
**

(a)

**
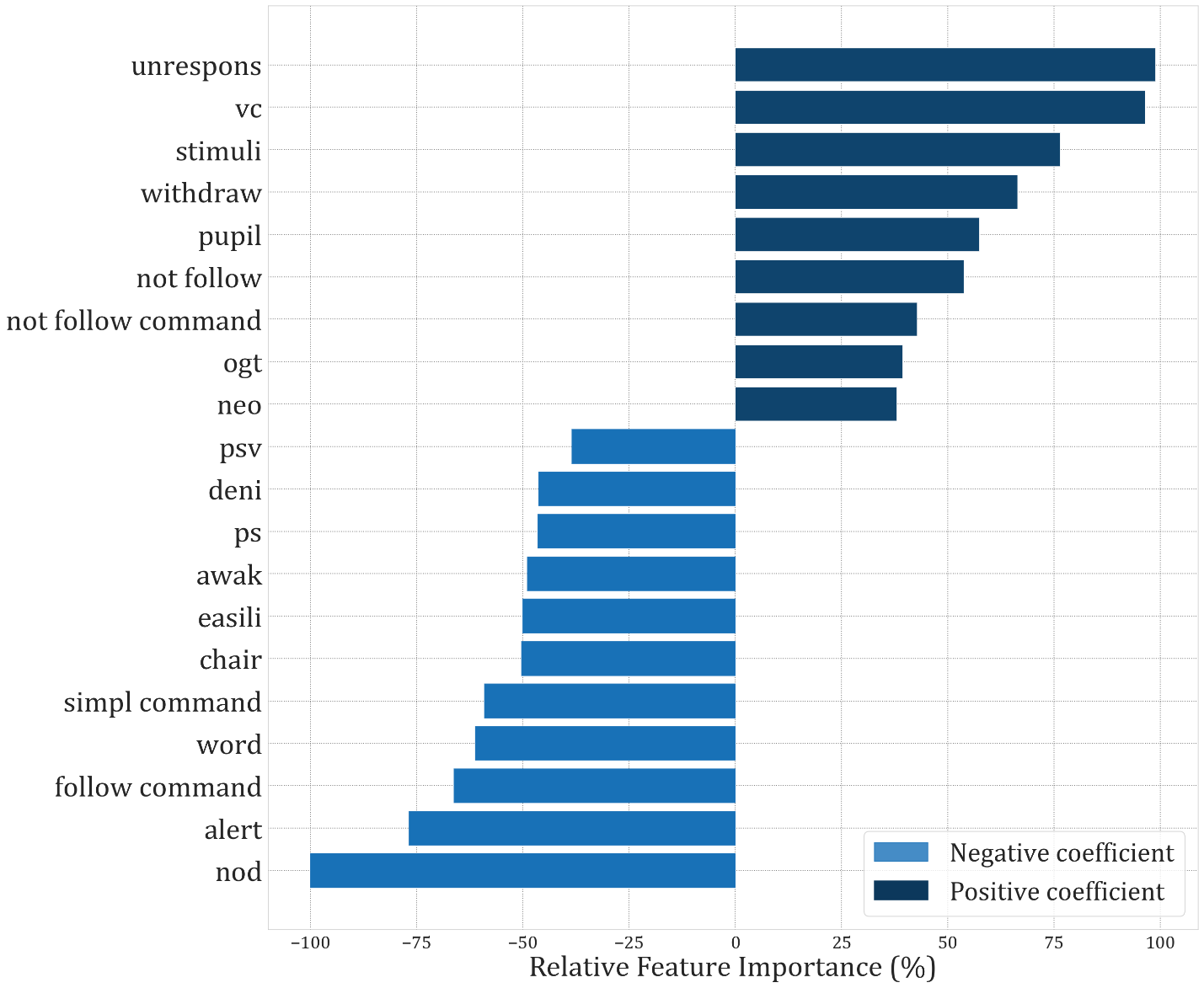
**

(b)

**Abbreviation legend:** Stemmed features derived from clinical documentation include airway and respiratory terms (ett, endotracheal tube; trach, tracheostomy; vent, mechanical ventilation; peep, positive end-expiratory pressure; ogt, orogastric tube; suction; carina; psv/ps, pressure support ventilation; vc, vital capacity), neurologic examination terms (unrespons, unresponsive; stimuli; withdraw, withdrawal to pain; pupil; not follow/not follow command; follow command; simpl command, simple commands; word, verbal response; nod; open eye; grimac, grimacing; agit, agitation; somnol, somnolence; awak, awake; alert; alert orient, alert and oriented; ox, derived from "A&Ox" [alert and oriented x]), and documentation/context terms (unabl assess, unable to assess; dnr, do not resuscitate; neo, phenylephrine/vasopressor; deni, denies; chair, mobilized to chair).

**Fig. S10 Confusion matrices of the pooled ordinal sensitivity model on the hold-out (a) full test set (b) MGB (c) MIMIC after calibration**

**
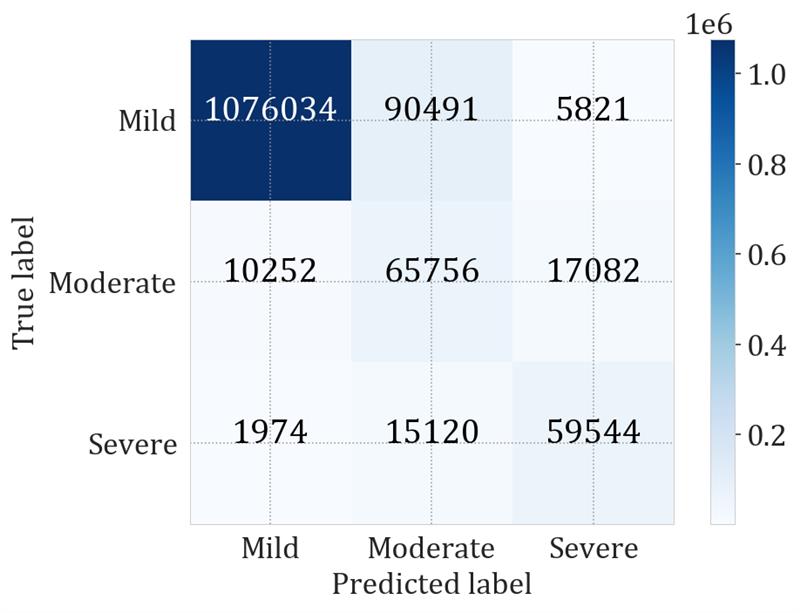

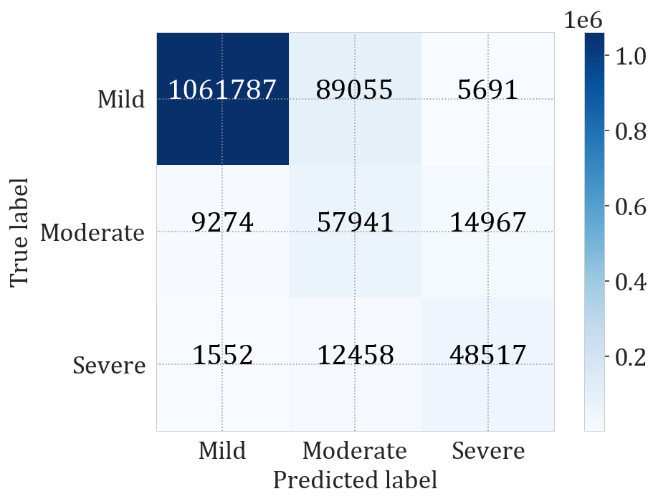
**

(a) (b)


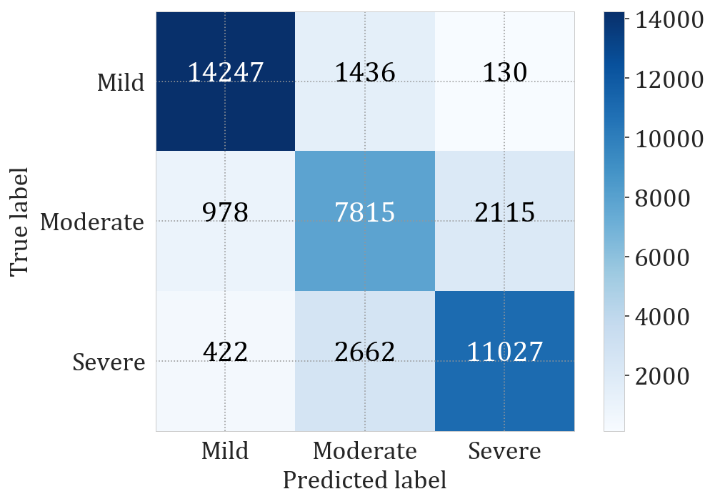


(c)

**Fig. S11 Calibration of the sensitivity model ordinal class probabilities on the hold-out test set: institution-specific reliability diagrams (a) MGB (b) MIMIC**


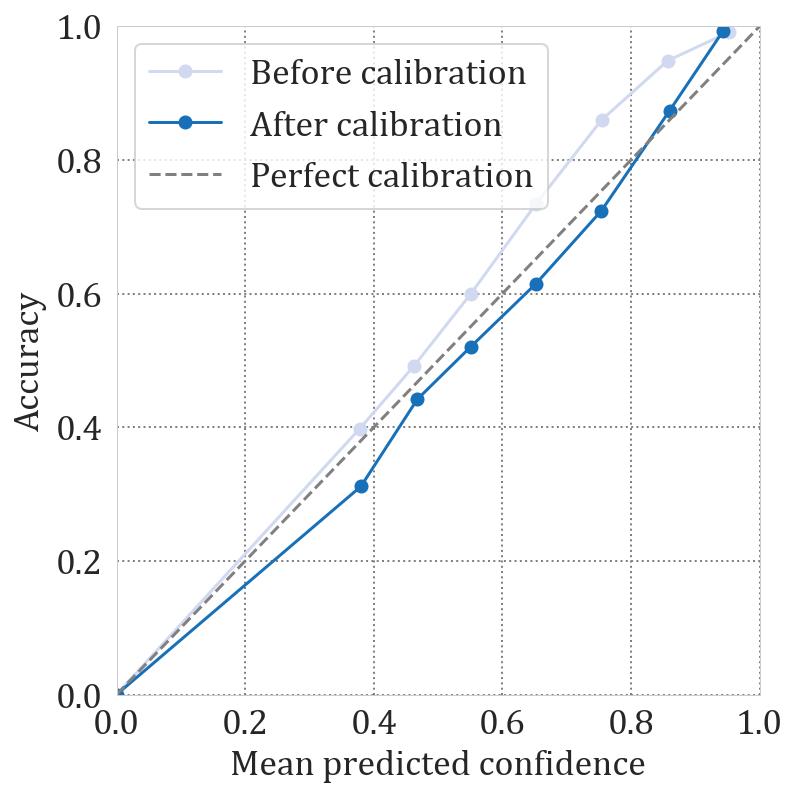

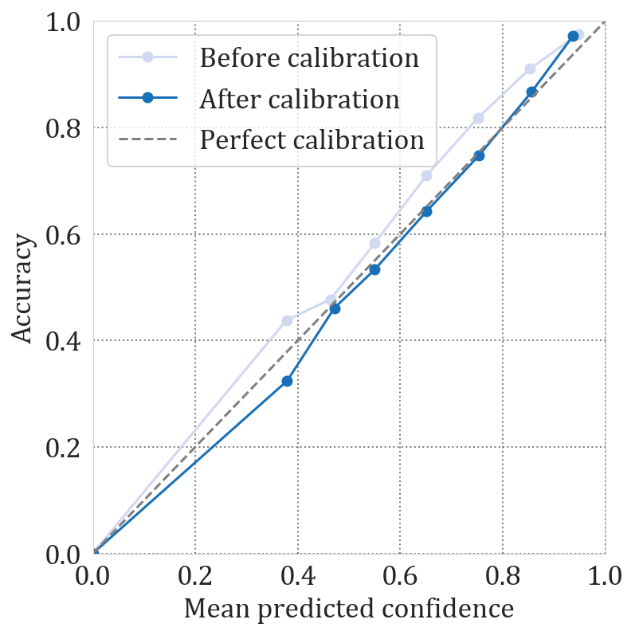


1. (b)

**References**

1. Porter MF. An algorithm for suffix stripping. Program. 1980 Jan 1;14(3):130–7.

2. Lemaître G, Nogueira F, Aridas CK. Imbalanced-learn: a python toolbox to tackle the curse of imbalanced datasets in machine learning. J Mach Learn Res. 2017 Jan 1;18(1):559–63.

3. Wurm MJ, Rathouz PJ, Hanlon BM. Regularized Ordinal Regression and the ordinalNet R Package. J Stat Softw. 2021 Sep;99(6):10.18637/jss.v099.i06.

4. Tibshirani R. Regression Shrinkage and Selection via the Lasso. J R Stat Soc Ser B Methodol. 1996;58(1):267–88.
